# Stable solar water splitting with wettable organic-layer-protected silicon photocathodes

**DOI:** 10.1038/s41467-022-32099-1

**Published:** 2022-08-01

**Authors:** Bo Wu, Tuo Wang, Bin Liu, Huimin Li, Yunlong Wang, Shujie Wang, Lili Zhang, Shaokun Jiang, Chunlei Pei, Jinlong Gong

**Affiliations:** 1 School of Chemical Engineering and Technology, Tianjin University, Tianjin, 300072 China; 2 Key Laboratory for Green Chemical Technology of Ministry of Education, Tianjin University, Tianjin, 300072 China; 3 Purification Equipment Research Institute of Handan, Handan, Hebei 056000 China; 4 Joint School of National University of Singapore and Tianjin University, International Campus of Tianjin University, Binhai New City, Fuzhou, 350207 China

**Keywords:** Electrocatalysis, Chemical engineering, Photovoltaics

## Abstract

Protective layers are essential for Si-based photocathodes to achieve long-term stability. The conventionally used inorganic protective layers, such as TiO_2_, need to be free of pinholes to isolate Si from corrosive solution, which demands extremely high-quality deposition techniques. On the other hand, organic hydrophobic protective layers suffer from the trade-off between current density and stability. This paper describes the design and fabrication of a discontinuous hybrid organic protective layer with controllable surface wettability. The underlying hydrophobic layer induces the formation of thin gas layers at the discontinuous pores to isolate the electrolyte from Si substrate, while allowing Pt co-catalyst to contact the electrolyte for water splitting. Meanwhile, the surface of this organic layer is modified with hydrophilic hydroxyl groups to facilitate bubble detachment. The optimized photocathode achieves a stable photocurrent of 35 mA/cm^2^ for over 110 h with no trend of decay.

## Abstract (web summary)

Preparation of inorganic protective layers for photoelectrodes requires high-quality deposition techniques. Here, the authors report a spin-coated organic protective layer that enables Si photocathodes to realize stable solar water splitting.

## Introduction

Converting solar energy into chemical fuels, such as hydrogen, is a sustainable way to relieve energy and environmental issues^1^. Silicon (Si)^2^, various metal oxides^3^, and III-V semiconductors^4,5^ have been studied for photoelectrochemical (PEC) water splitting. Among them, Si is one of the most attractive candidates for PEC hydrogen evolution reaction (HER) due to its low cost, suitable bandgap, and band edge position^6^. However, Si is susceptible to deactivation in aqueous solutions^7–9^. To construct a stable Si-based photocathode, the prevailing strategy is to introduce a compact metallic layer or metal oxide layer by sputtering^10^ or atomic layer deposition (ALD)^11^ on the Si surface to isolate Si from the electrolyte. Thus, this compact protective layer is required to be transparent^12^, conductive^13^, and chemically stable^14^ to enable a durable and efficient photocathode^15^. TiO_2_ films formed by ALD have been extensively employed as protective layers for PEC electrodes^16–18^. However, TiO_2_ will become unstable at very negative potentials^19^ or under UV illumination^20^. Meanwhile, previous studies found that ALD-grown TiO_2_ required a minimum thickness of 50 nm to become pinholes-free in a strict sense, due to the presence of atmospheric particulate matter on the substrate in a normal laboratory environment^21–25^. Additionally, pitting corrosion corresponded to pinholes was also demonstrated^26^. Thus, the traditional isolation strategy that requires a pinholes-free layer imposes a limitation on material selection and deposition method. Therefore, there is a need to develop strategies^27^ that could diminish Si-liquid contact with acceptable tolerance for pinholes in the protective layer.

In addition to isolating Si substrate from electrolyte with a dense protective layer, it is also possible to decrease the Si-liquid contact by increasing the hydrophobicity of Si to delay the transport of liquids near the surface^28^, which might decrease the rate of corrosion^29,30^. However, decreasing Si-liquid contact will also diminish the active area for water splitting reactions. Moreover, previous studies observed that most hydrophobic layers were poorly conductive^31,32^, while hydrophobic electrodes exhibited vastly lowered HER activities than wettable electrodes^33,34^. The poor performance of hydrophobic electrodes could be attributed to the fact that a large amount of gas would grow into big bubbles on the surface during the HER^35–37^, which would cause a current drop due to the covering of active sites of the HER catalysts^38^. Therefore, it is highly desired but still challenging to use a hydrophobic layer as the protective layer while preventing the bubble covering over co-catalysts to achieve efficient surface reactions.

This study describes the design and fabrication of a discontinuous protective layer with controllable surface wettability, where a bottom organic hydrophobic layer is modified with surface hydrophilic hydroxyl groups to form thin gas layers to isolate the electrolyte from the corrosion-vulnerable Si substrates while suppressing the formation of big bubbles. By optimizing the pore sizes of the hydrophobic layer, discrete thin gas layers will form in the pores to isolate the exposed Si from the corrosive solutions, while allowing HER co-catalyst Pt to contact the electrolyte to maintain a high photocurrent. Under the protection of this organic layer deposited by a spin-coating method, the photocathode achieves a saturation current of 35 mA/cm^2^ with a stable operation for 110 h without performance deterioration, which is comparable to metal oxide-based protective layers prepared by vacuum deposition techniques.

## Results

### PEC performances of Si-photocathodes with the organic protective layers

**Fig. 1:**
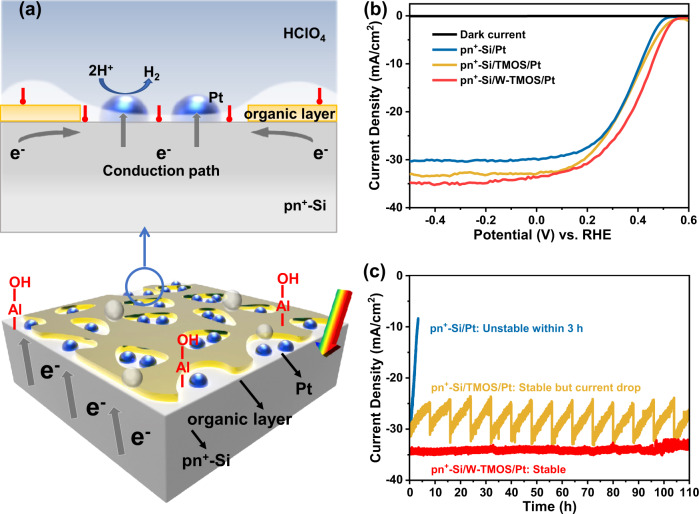
PEC performance of photocathodes protected by organic protective layers. **a** Electron conduction path of pyramid pn^+^-Si/organic layer/Pt photocathode. **b** J–V curves of photocathodes with different protective layers under AM 1.5 G illumination. **c** Stability tests of photocathodes with different protective layers at 0 V vs. RHE.

For electrode fabrication (details in Fig. S1), an ordinary pyramid pn^+^-Si substrate (Fig. S2) was used as a model sample to establish the relationship between performance and surface wettability, which exhibits a stable and reproducible photovoltage of 0.55 V. It is known that organosilane, such as Si(O-CH_3_)_3_-(CH_2_)_17_-CH_3_ (TMOS), presents a suitable surface tension^39,40^. At the same time, the thickness of organosilane can be controlled on a nanometer scale, with structures varied from self-assembly monolayer to multilayer by changing the deposition conditions^41^. Furthermore, the structure or surface functional group of organosilane is sensitive to temperature^42,43^. Thus, it is possible to fabricate an organosilane layer with a controllable thickness and structure to enable efficient electron transport. Therefore, TMOS is selected to increase the hydrophobicity of the substrate. By optimizing the speed of spin-coating and the temperature of hydrolysis and condensation steps, TMOS could be feasibly modified to a discontinuous layer with controllable pore sizes (200 nm to 1 µm), while the thickness is maintained at around 5 nm (thickness was obtained from the planar p^++^-Si/TMOS sample) to achieve a high photocurrent with high stability (Figs. 1a, S2 and S3). The nanoporous morphology of the solution-processed TMOS layer may be closely related to the removal of ethanol and water during the 120 °C curing process^44^. The side view scanning electron microscopy (SEM) images show that there is no difference between the pyramid pn^+^-Si and pn^+^-Si/TMOS samples (Fig. S4), revealing that the TMOS layer will not coalesce together, nor accumulate at the bottom of the pyramid arrays. Even though the ultrathin TMOS film cannot be directly observed on the Si pyramid from side view SEM (Fig. S4) or cross-sectional transmission electron microscopy (TEM) (Fig. S5) images, it is still possible to conclude the existence of a surface layer on the Si pyramid surface. The top view energy dispersive spectroscopy (EDS) mapping of the pn^+^-Si/TMOS/Pt shows that the C element is distributed evenly upon the pyramid pn^+^-Si. At the same time, the content of the C element is increased to 24.4% from 2.3% upon the introduction of TMOS layer (Fig. S6). Furthermore, the contact angle is increased to 110° from 65° (Fig. S2). These results confirm that the TMOS layer indeed exists on the pyramid surface.

With homogeneously distributed single clusters of Pt (8 s sputtering, details in SI) as the HER co-catalyst (Figs. S6–S8), which lowers the activation energy (overpotential) for the HER^45^. The current-potential (J–V) characteristics show that the optimized pn^+^-Si/TMOS/Pt exhibits an improved PEC activity compared with the pn^+^-Si/Pt sample, which may be due to the decreased charge recombination upon TMOS passivation (Figs. 1b and S9). Specifically, the pn^+^-Si/TMOS/Pt photocathode achieves a saturation photocurrent density of 32.5 mA/cm^2^. Apart from the better PEC activity, the pn^+^-Si/TMOS/Pt photocathode exhibits much stronger stability. The photocurrent of unprotected pn^+^-Si/Pt photocathode decays quickly within 3 h due to the formation of SiO_2_ (Figs. 1c and S10)^20^. Photocathode with the structure of pn^+^-Si/TMOS/Pt shows almost 22% decreased photocurrent after 8 h. However, it can recover its original photocurrent after being taken out of the electrolyte and put back immediately to remove the accumulated gas bubbles, leading to stability for over 110 h by repeating this bubble removal operation every 8 h (Fig. 1c). Similar current drops during the stability tests were also observed in previously reported photocathode with a structure of pn^+^-Si/Ti/TiO_2_/Pt or pn^+^-Si/MoS_2_^46,47^. However, the mechanisms might be different. Previous studies attributed the decrease of the photocurrent to the contamination of Pt co-catalysts^46^ or the elevated temperature^47^. Therefore, co-catalysts or electrolytes were replaced to recover the original performance. However, no extra treatments were needed in this study. The photocathode was simply taken out of the electrolyte, and then submerged into the electrolyte immediately (within 60 s) every 8 h during the stability test. The photocurrent can reconvert to the original level, by which it can be inferred that bubble accumulation is the reason for the photocurrent drop in our case.

To eliminate the current drop caused by bubble accumulation, trimethylaluminum (TMA) molecules were introduced on the surface of the hydrophobic layer to alter the surface wettability of the TMOS layer. Al 2p peak appeared in the X-ray photoelectron spectroscopy (XPS) spectrum of the pn^+^-Si/W-TMOS sample, which demonstrates that TMA adsorbed onto the TMOS layer (Fig. S11)^48^. As a result, a wettable TMOS layer (denoted as W-TMOS) can be obtained after ambient exposure, where the water molecules from the ambient will convert surface adsorbed TMA into -Al-OH groups (without H_2_O dose in the ALD chamber), leaving a -OH terminated hydrophilic surface (Figs. S12 and S13). It should be noted that this surface treatment only involves the repeated dosing of TMA without dosing water, which is different from ALD processes. The optimized cycle number of TMA dosing is determined according to the photocathode performance, which presents a volcanic trend with the increasing cycle numbers (Fig. S14). Photocathode pn^+^-Si/W-TMOS/Pt achieves a saturation photocurrent density of 35 mA/cm^2^ with an applied bias photo-to-current efficiency (ABPE) of 8%, surpassing pn^+^-Si/TMOS/Pt and pn^+^-Si/Pt photocathodes (Fig. S15). This PEC performance is also comparable to other metal oxides protective layers-based photocathodes (Table S1). The high efficiency of pn^+^-Si/W-TMOS/Pt photocathode is supported by much higher incident photon-to-current efficiency (IPCE), as compared to that of pn^+^-Si/TMOS/Pt (Fig. S16a). To investigate the optical effect of the samples, a calibrated photodiode (Thorlabs, FDS100-CAL) was used to measure the intensity of reflected light from the pyramid Si samples with a different protective layer and different gas evolving conditions in the electrolyte (details in SI, Fig. S16d). The result shows that the hydrophobic TMOS layer induces additional reflective interfaces, with the reflectivity increasing to 10.2% from 7.8% compared to bare Si. Furthermore, the accumulated large bubbles on the TMOS layer will further increase the reflectivity to 13.7%. On the contrary, the hydrophilic W-TMOS sample exhibits a reduced reflectivity to 4.7%, and the small bubbles on the pyramid pn^+^-Si/W-TMOS/Pt surface only bring an extra reflectivity of 0.9% (Figs. S16b, c). Thus, the enhanced IPCE obtained by pyramid pn^+^-Si/W-TMOS/Pt over pn^+^-Si/TMOS/Pt might be attributed to the suppressed light reflectivity owing to the hydrophilic surface that suppresses the accumulation of gas bubbles. The Faradaic efficiency for H_2_ of pn^+^-Si/W-TMOS/Pt photocathode is above 97%, indicating the efficient conversions of photoelectrons to H_2_ (Fig. S17). All PEC results indicate that the optimized W-TMOS and TMOS layers will not affect the electron transfer. Furthermore, the PEC performances demonstrate that optimized W-TMOS and TMOS layers do not affect the light transmittance. Ultraviolet-visible (UV-vis) spectroscopy results show that TMOS and W-TMOS layers are transparent at the optimized thickness of 5 nm. However, 50 nm TiO_2_, the thickness required for a pinholes-free layer, absorbs at least 20% of the incident light at 200–800 nm wavelength (Fig. S18). Additionally, the pn^+^-Si/W-TMOS/Pt photocathode achieves stability above 110 h (Figs. 1c, S19 and S20). Under the simulated realistic operational condition (8 h simulation and 16 h dark, repeated), the optimized pyramid pn^+^-Si/W-TMOS/Pt also achieves long-term stability above 110 h, which demonstrates that this surface modification method is effective on materials subjected to intermittent illumination (details in SI). These results reveal that the facile spin-coating-based organic protective layer indeed achieves long-term stability, which is comparable to the inorganic protective layer deposited by vacuum deposition techniques.

### Electron transportation through the organic protective layer

**Fig. 2:**
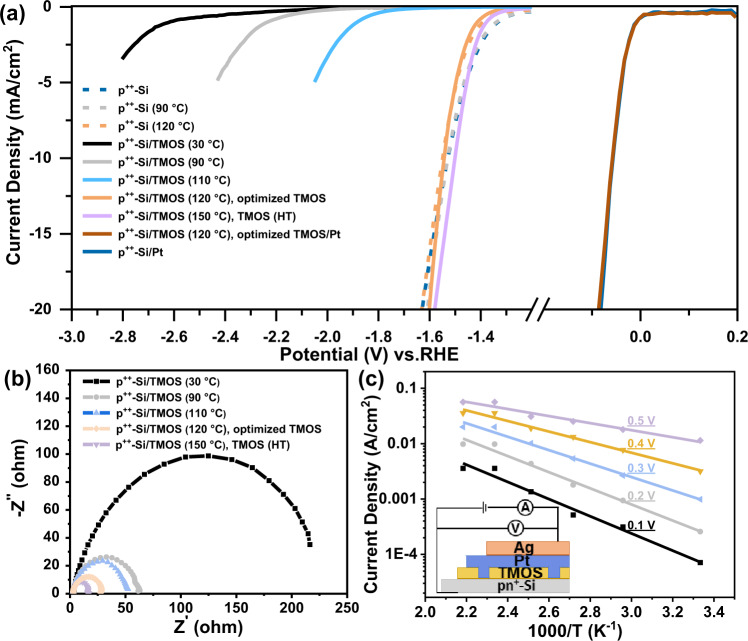
Impacts of TMOS baking temperature on electron transfer efficiency. **a** J–V curves of planar p^++^-Si with different treatments. **b** Nyquist plots of planar p^++^-Si/TMOS formed under different temperatures at −1.4 V vs. RHE. **c** Current-temperature curves of pyramid pn^+^-Si/TMOS/Pt sample at different applied voltages, from dark solid J–V measurements (Inset: Schematic illustrations of the solid-state device for the dark solid J–V measurements).

TMOS is a poorly conductive material^31,32^. Furthermore, the thickness of the TMOS layer is about 5 nm, which is too thick to allow electrons to tunnel through^49^. Thus, the electron transport mechanism through this organic layer needs to be further explored. The optimization process indicates that the formation temperature of TMOS layer largely affects the performances of photocathodes. To further investigate the electron transfer pathway across these organic layers, TMOS were deposited at various temperatures (30–150 °C) on planar p^++^-Si instead of the pyramid pn^+^-Si. The planar substrate provides a simplified structure of thin organic layers with a thickness on the nanometer scale. A dummy solution (ethanol and water only) without TMOS was also spin-coated onto the planar p^++^-Si surface to form a control sample. After that, these electrodes were heated under 90 °C and 120 °C, respectively. The control samples were denoted as p^++^-Si (90 °C) and p^++^-Si (120 °C). The p^++^-Si (90 °C), p^++^-Si (120 °C), and p^++^-Si samples exhibit similar onset potential and slope for the HER, which demonstrates that the baking temperature will not induce the oxidation of the Si surface (Fig. 2a). J–V tests show that samples of planar p^++^-Si/TMOS (below 110 °C) present low current density. However, TMOS film heated under 120 °C or 150 °C exhibits similar performance with planar p^++^-Si, which indicates that temperature can increase the electron transfer efficiency. The optimized TMOS layer discussed below was deposited at 120 °C, while the TMOS layer formed under a higher temperature (150 °C) is denoted as TMOS (HT). Additionally, electrochemical impedance spectroscopy results show the smaller arc radius of p^++^-Si/TMOS samples baked under higher temperatures, which also proves that the electrodes formed under higher temperatures show faster electron transfer (Fig. 2b).

**Fig. 3:**
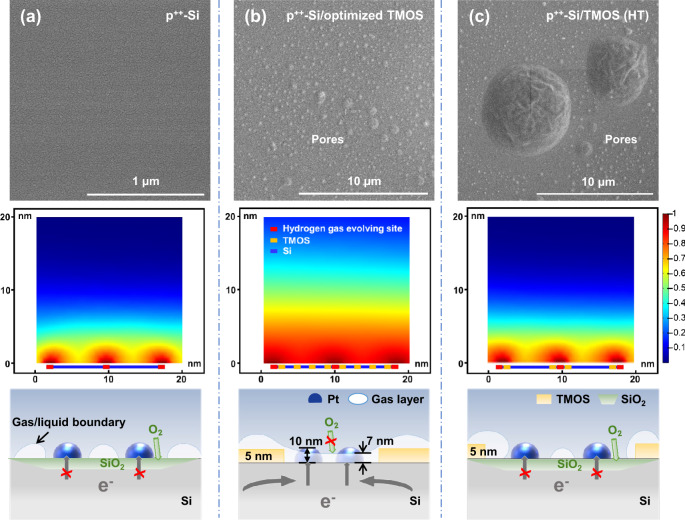
Protective mechanism of organic protective layers with different pore sizes. SEM images, simulation results of Si-liquid contact, and the corresponding schematic illustrations of electron conduction path (not to scale) for **a** Si, **b** Si/TMOS, and **c** Si/TMOS (HT). Middle row: the color bar shows the volume fraction of H_2_ gas in the liquid/gas mixture in CFD simulation, in which the red color in the simulation result (second row) represents an H_2_ volume fraction of 100% (electrolyte 0%), while the blue color represents an H_2_ volume fraction of 0% (electrolyte 100%).

Quantitative in situ diffuse reflectance infrared Fourier-transform spectroscopy (DRIFTS) and Raman spectroscopy did not show any evidence of conductive functional groups^50^ or conductive materials^51^ formed in Si/TMOS at elevated temperature (Fig. S21). These results reveal that increased temperature does not change the chemical structure of TMOS. Thus, increased temperature makes no noticeable influence on the intrinsic conductivity of the TMOS layer. SEM image indicates increased pore size and density with increasing temperature (Fig. 3). The atomic force microscopy (AFM) images also reveal that there are some pores in the TMOS layer (Fig. S22). That is because high temperatures would accelerate the hydrolysis and condensation of TMOS, making it a disordered film^42^. Therefore, it could be concluded that the main difference between TMOS layers formed under different temperatures is the size and density of the pores, which may influence the transfer of electrons.

SEM results show that the optimized pore sizes of the TMOS layer are ~1 µm, while the optimized sizes of Pt particles are ~10 nm (Fig. S7). Therefore, the Pt particles can contact Si in the pores of the TMOS layer. Furthermore, the measured photogenerated minority carrier diffusion length of pn^+^-Si substrate used in this study is above 117 µm, while the distance between adjacent pores in the TMOS layer is less than 10 µm. This makes it possible for electrons generated under covered Si to transfer laterally to exposed Si at pores, where Pt particles contact Si for the HER. The cyclic voltammogram result shows that the pyramid pn^+^-Si/TMOS/Pt electrode exhibits a much higher redox peak current than pn^+^-Si/TMOS (Fig. S24a). The J–V curves also show that the onset potential shifts positively by about 1 V upon the deposition of Pt particles when H^+^/H_2_ or aqueous methyl-viologen was adopted as the redox couple (Figs. S24b and S25b). These results demonstrate that optimized Pt particles enhance the electron transfer across resistive TMOS interfaces by forming a conduction path of Si/Pt/electrolyte^52–55^. The open circuit potential versus time of pyramid pn^+^-Si/TMOS/Pt photocathode in the dark and under AM 1.5 G illumination shows that the photovoltage of pyramid pn^+^-Si/TMOS/Pt photocathode is about 0.55 V, which is similar with the voltage obtained by pyramid pn^+^-Si/TMOS (Fig. S25c). This result reveals that Pt deposition will not pin the band edges of Si to the Si/Pt defect site and the photovoltage is primarily determined by the pn junction. Furthermore, J–V curves of pyramid pn^+^-Si/TMOS/Pt photocathode under different solutions with different pH show that the V(onset) values do not change with pH (Fig. S25a). However, when the aqueous H^+^/H_2_ is changed to aqueous methyl-viologen as a redox couple, the V(onset) shifts negatively from 0.55 V to ~0.25 V (Figs. S25b and S26b). These results demonstrate that the Pt deposition will not result in the Fermi level pinning.

Dark solid J–V measurements of pn^+^-Si/TMOS/Pt sample at various temperatures show an ohmic contact conduction mechanism (Figs. 2c, S23, and Table S2), which confirms that electrons transfer through Si/Pt interface directly. Furthermore, J–V curves of pyramid pn^+^-Si/TMOS/Pt show that photocathodes protected by TMOS with different chain lengths (12, 16, or 18 carbons, denoted as 12 C, 16 C, or 18 C) exhibit similar PEC performance (Fig. S26a). This is due to the fact that the polymer based on 12 C or 16 C remains poorly conductive since it does not contain any conductive functional group or conductive materials. The electrons cannot transfer across these resistive interfaces, leaving Si/Pt contacts in the pores as the only electron transfer pathway. However, polymers with a shorter chain length exhibit reduced photocathode stability (Fig. S26b). Thus, it could be concluded that the promoted electron transfer efficiency is indeed due to the formation of the Si/Pt ohmic contacts in the larger pores of the TMOS layer formed under high temperatures. Apart from the enhanced electron transfer with the Pt deposition, Pt particles lower the kinetics barrier for the HER. J–V curves show that pyramid pn^+^-Si/TMOS/Pt achieves a steeper slop than pn^+^-Si/TMOS, when aqueous H^+^/H_2_ is adopted as that redox couple. This result is also consistent with the J–V curves of planar p^++^-Si/TMOS and p^++^-Si/TMOS/Pt (Fig. 2a). Moreover, planar p^++^-Si/TMOS/Pt and p^++^-Si/Pt electrodes show the same onset potential and current density (Fig. 2a). This result may demonstrate that the optimized Si/TMOS/Pt exhibits sufficient active site for the HER by forming Si/Pt contact in the pores of the discontinuous layer.

### Protection mechanism of the discontinuous organic protective layer

It is generally accepted that pinholes in inorganic hydrophilic protective layers, such as oxides, would lead to a decreased stability due to the exposed Si substrate contacting corrosive electrolyte directly^46^. However, the photocathode protected by the optimized discontinuous organic hydrophobic protective layer exhibits great stability in this study. Thus, a different protection mechanism is proposed by which the discontinuous TMOS protects the Si with a finely controlled gas layer formation process. The covered part of Si will be protected by TMOS, while it is suspected that the exposed Si might be isolated from corrosive solution through the formation of thin gas layers, while the gas layer should be thin enough to allow Pt particles to penetrate through this layer to contact with the electrolyte for a large photocurrent.

To prove this hypothesis, computational fluid dynamic simulations (CFD) were used to analyze the solid-liquid-gas contact state for different electrodes. The simulation result reveals the formation of a continuous gas layer above the surface of the hydrophilic layer (Fig. S27). This result indicates that exposed Si can be protected by the continuous gas layer. At the same time, the CFD result on the nanometer scale shows that a thin gas layer, with a thickness <7 nm, forms above the surface of the exposed Si on the planar Si/TMOS electrode. This result reveals that the Pt nanoparticles with a size of 10 nm can penetrate through this gas layer to contact the electrolyte for a large photocurrent. However, the exposed Si will contact the electrolyte directly for planar Si without the protection of the TMOS layer (Figs. 3b and S27). In other words, the thin gas layer may protect Si from corrosion without affecting the HER. Furthermore, the simulation result also demonstrates the influence of the wettability of exposed Si on the formation of the thin gas layer at the micrometer scale (Figs. S27 and S28), where the contact angle of the exposed Si was decreased to 50° from 65° (Fig. S2). The simulation result shows that the increased wettability of the exposed Si at pores will not affect the formation of the continuous gas layer, when the pore sizes of the TMOS remain constant (Fig. S28). The result demonstrates that the thin gas layer is also the reason for the long stability of the pn^+^-Si/W-TMOS/Pt photocathode. However, when the electrodes are not coated by the TMOS layer or when the pore size is larger than 5 µm, simulation results show that Si contacts the electrolyte directly (Figs. 3a, c). Thus, the Si will be corroded, which is the main reason for the poor stabilities of pn^+^-Si/Pt and pn^+^-Si/TMOS (HT)/Pt photocathodes.

### Impact of bubbles on the performance drop

**Fig. 4:**
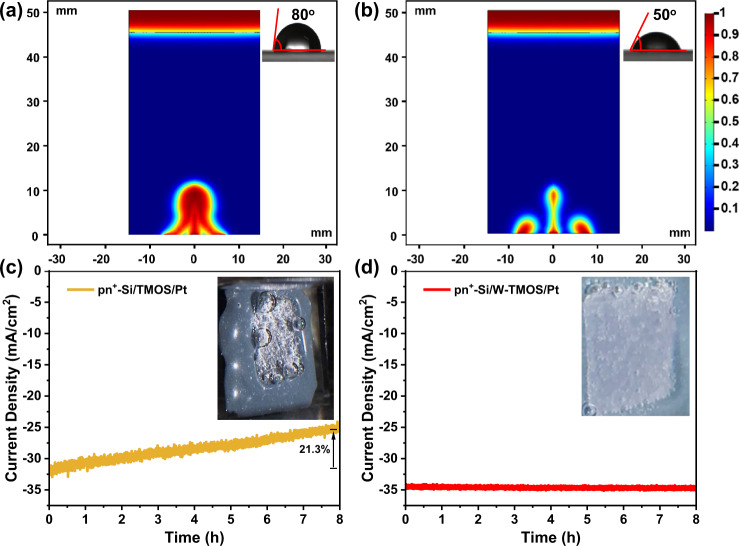
Bubble evolution process and current drop on organic TMOS protective layers. Simulation results of bubble growth of **a** Si/TMOS, and **b** Si/W-TMOS electrode. (Inset: Contact angle). The color bar shows the volume fraction of H_2_ gas in the liquid/gas mixture. Stability test of **c** pn^+^-Si/TMOS/Pt, and **d** pn^+^-Si/W-TMOS/Pt at 0 V vs. RHE. (Inset: electrode picture during the stability test).

Though TMOS and W-TMOS coated photocathodes share the same protection strategy, they exhibit drastically different bubble growth kinetics, which attributes to their different surface wettability. The contact angle of pn^+^-Si/TMOS/Pt electrode is 80° (Inset of Fig. 4a). Thus, as-formed gas adheres strongly to the TMOS layer. It was found that only large bubbles were released from the electrode surface while the smaller bubbles continued to grow during the HER (Movie S1 and inset of Fig. 4c). Furthermore, the simulation result indicates that bubbles coalesce with adjacent bubbles before detachment (Figs. 4a and S29). Such growth and release behaviors of the large bubbles result in decreased effective reaction areas. Therefore, while photocathode protected by TMOS allows the possibility of achieving long-term stability, it suffers from the current drop caused by the bubble accumulation (Fig. 4c).

The situation result is drastically different when W-TMOS is used as a protective layer. With the modification of hydrophilic hydroxyls groups on the top of TMOS, the surface of pn^+^-Si/W-TMOS/Pt becomes more hydrophilic, with the contact angle decreased to 50° (Inset of Fig. 4b). It was observed that the pn^+^-Si/W-TMOS/Pt photocathode exhibited a quick bubble detachment during the stability test (Movie S2 and inset of Fig. 4d), which is consistent previous work^56^. Additionally, the simulation result proves that the bubble growth is limited when the wettability is increased (Fig. 4b), which leads to high performance with long-term stability (Fig. 4d)^57^. Therefore, the wettable organic layer will isolate the electrolyte from the corrosion-vulnerable substrates, while accelerating the detachment of gas bubbles. Consequently, a stable and effective Si-based photocathode can be constructed.

## Discussion

A facile and reliable method for resolving the trade-off between efficiency and stability of the organic protective layer is demonstrated by constructing a discontinuous organic layer with adjustable surface wettability. By optimizing the pore size of the organic layer, the discrete thin gas layers will form in the pores to isolate the exposed Si from the electrolyte to achieve long-term stability, while Pt co-catalyst penetrates through the gas layer to contact the electrolyte to maintain a high photocurrent. Furthermore, the hydrophilic W-TMOS layer also decreases the light reflectivity by suppressing bubble accumulation compared with a hydrophobic surface. Under the protection of W-TMOS layer, the Si photocathode achieves a high ABPE of 8% in strongly acidic electrolyte without any degradation over a duration of 110 h. The relationship between the wettability of the photoelectrode and performance in this work may provide a general strategy for altering the instability and bubble problems of photocathodes for the HER. Furthermore, more research endeavors might be needed to investigate the relationship between the thickness of the organic layer and the PEC performance.

## Methods

### Fabrication of pyramid pn^+^-Si

p-Si(100) used for the fabrication of pyramid pn^+^-Si(100) was obtained from Hefei Kejing Material Technology Co., Ltd. The pyramidal surface textured (pyramid) pn^+^-Si substrate with a pyramid height of around 300~350 nm (Fig. S22b) was produced by the KOH etching due to the anisotropic corrosion between the <100> and <111> planes of Si substrate. After etching, the buried pn junction was fabricated by a thermal diffusion technique, using a phosphorus liquid source of phosphorous oxychloride (POCl_3_). Detailed procedures:

First of all, the Si(100) wafer was cleaned with a Radio Corporation of America (RCA) cleaning method to remove the attached particles, organic matters, and metal ions from the surface of the wafer. The pyramid Si was then fabricated through anisotropic etching in a mixture solution of potassium hydroxide (KOH) (6 wt%) and isopropanol (3 vol%) at 80 °C for 25 min. After that, the sample was removed and cleaned again as in the first step. Then, the wafer was rinsed with high purity water and blow-dried with nitrogen. After the wet etching method, 400 μL/cm^2^ dopant (POCl_3_) was spun on the silicon front surface at a speed of 3500 rpm for 45 s. Dopant diffusion and activation were conducted at 900 °C with a tube furnace for 30 min. The pn^+^-Si wafers were then etched for 30 s in hydrofluoric acid solution (BHF) to remove the formed dopant oxide.

The highly doped planar p^++^-Si was obtained from Hefei Kejing Material Technology Co., Ltd, which was used as the conductive substrate.

### Modification of TMOS layer

The pyramid pn^+^-Si was dipped in 1% HF for 3 min to remove the native SiO_2_ layer. At the same time, 4% TMOS (vol%) (Shanghai Aladdin Industrial Co., Ltd), 86% ethanol (vol%) (Tianjin Jiangtian Chemical Technology Co., Ltd), and 10% water (vol%) (18.25 MΩ ∙ cm), supplied by a UP Water Purification System) were mixed into a highly dispersed emulsion by putting into an ultrasonic cleaner (Wiggens, UA10MFDN). After that, the TMOS precursor was spin-coated onto the surface of pn^+^-Si at a speed of 5500 rpm. Then, the electrode was heated under 120 °C for 5 min to accelerate the hydrolysis and condensations of the TMOS layer.

### Adsorption of TMA

5 cycles of water were dosed on the TMOS surface firstly to maximize the initial hydroxyl sites. After that, repeated dosing of TMA (Suzhou Fornano Electronics Technology Co., Ltd) was performed without dosing water, which is different from the typical ALD process, to achieve controllable surface wettability. One dosing cycle consists of TMA dose for 0.05 s, followed by an N_2_ purge for 10 s.

### Loading of Pt nanoparticles

Pt co-catalysts were deposited via DC magnetron sputtering for different durations. Before deposition, the chamber was evacuated to 10^−5^ Pa, and then a high purity Ar flow was introduced into the chamber. The gas flow was fixed at 20 sccm by a mass flow controller. The working pressure was fixed at 1 Pa and the deposition time is regulated to achieve the desired film thickness. The optimized deposition amount is 0.075 mg/cm^2^.

### Characterization

The morphology and microstructure of the samples were characterized by field emission scanning electron microscopy (FE-SEM, S-4800, Hitachi, 3–5 kV), transmission electron microscopy (TEM, JEM-2100F, JEOL, 200 kV), and atomic force microscope (AFM, Bruker, Dimension icon). XPS of the samples were carried out on a Physical Electronics PHI 1600 ECSA system with an Al Kα X-ray source (E = 1486.6 eV). The binding energy was calibrated against the C 1 s photoelectron peak at 284.6 eV as the reference.

The transmission spectra of samples were obtained by the UV-vis spectrophotometer (Shimadzu, UV-2550).

The in situ DRIFTS experiments were performed on a Thermo Scientific Nicolet IS50 spectrometer, equipped with a Harrick Scientific DRIFTS cell fitted with ZnSe windows and a mercury-cadmium-telluride (MCT) detector cooled by liquid nitrogen. The backgrounds (8 cm^−1^ resolution, 64 scans) were collected with a two-side polished Si, which was treated by HF, after Ar purging in a flow rate of 20 sccm for at least 30 min. After the baseline measurement, the DRIFTS spectra were collected under different temperatures upon the baseline subtraction, during which a mixture of 20 sccm Ar and 10 sccm Air was used as the purging gas.

The Raman spectroscopy of the TMOS layer was performed with a Raman microscopy system (Horiba Jobin Yvon, LabRAM HR Evolution). A He-Ne laser (λ = 532 nm) served as the excitation source.

The thickness of TMOS layer was measured by a spectroscopic ellipsometer (J. A. Woollam, M-2000D).

### Wettability test

Wettability tests were conducted on a drop shape analysis system with a sessile drop method applied by a contact angle tester (Shanghai Zhongchen Digital Technology Apparatus Co., Ltd., Powereach JC2000C1) in ambient conditions at room temperature. The spreading of the water droplet with a volume of 10 μL on the sample surface over time was observed, while the wettability of the samples was estimated by observing the contact angle of the water droplet on the surface at the stable state.

### Temperature-dependent dark solid-state J–V measurements

The dark solid J–V curves were measured by the Keithley 2450 Source Meter. 100 nm Pt was deposited onto the TMOS surface. After that, 200 nm Ag was deposited, which was connected by a Cu wire as the back contact. Ohmic contacts to the semiconducting electrodes were formed by rubbing an In-Ga eutectic onto the backside of the Si samples for solid tests. The substrate bias was scanned from −1.0 V to 1.0 V while monitoring the current density under different temperatures. The J–V curves were collected continuously until no visible change was observed under different temperatures.

### PEC measurements

PEC measurements were performed in 1 M HClO_4_ (pH 0) using a quartz 3-electrode cell with prepared electrodes as the working electrode, Pt foil as the counter electrode, and Ag/AgCl as the reference electrode. A 300 W Xenon lamp (Beijing Perfectlight, PLS-SXE300C) equipped with an AM 1.5 G filter was used to simulate sunlight, and the power intensity of the light was calibrated to 100 mW/cm^2^ against a calibrated Si diode (Thorlabs). The potentials were rescaled to the potentials versus the reversible hydrogen electrode (RHE) according to Eq. (1):

1 $${{{\mbox{E}}}}_{{RHE}}\,\left({{\mbox{V}}}\right)={{{\mbox{E}}}}_{{Ag}/{AgCl}}{{\mbox{+}}}0{{\mbox{.}}}059\times {{\mbox{pH+}}}0{{\mbox{.}}}197$$

The J–V curves (without IR compensation) of the samples were measured with a scan rate of 50 mV/s under irradiation with the simulated AM 1.5 G irradiation. The active geometric areas of the working electrode were calibrated by the software ImageJ.

The ABPE of the photocathodes was calculated using the J–V curves with an assumption of 100% Faradaic efficiency, according to Eq. (2):

2 $${{{{{\rm{ABPE}}}}}}\,(\%){{\mbox{=}}}\frac{\vert {{{{{\rm{J}}}}}}|\times (1.23-{{{{{{\rm{V}}}}}}}_{b})}{{{{{{\rm{P}}}}}}}\times 100\%$$

Where J (mA/cm^2^) is the photocurrent density, V_*b*_ is the applied bias (V vs. RHE), and P is the incident illumination intensity (100 mW/cm^2^ in this work).

Faradaic efficiency test was carried out in a custom-designed air-tight H-type electrochemical cell (H-cell, Chinese patent, CN201410777273.7) with an illumination window in the sidewall of the reactor, through which illumination can be applied to the photocathode. The cathode was connected to a gas circulation system with a tenport value (VICI) for online sampling to a gas chromatograph (GC, Agilent, 7890B) for H_2_ gas product detection. Before the start of the reaction, both the electrolyte and circulation system were purged with N_2_ (≥ 99.995%) for 30 min to remove air. After that, continuous N_2_ with a flow of 30 sccm was purged to the electrolyte during the test. The Faradaic efficiency for H_2_ production of the Si photocathode was calculated according to Eq. (3):

3 $${{{{{{\rm{\varphi }}}}}}}_{F}{{\mbox{(}}}\%{{\mbox{)=}}}\frac{{{{\mbox{Q}}}}_{{\exp }}}{{{{\mbox{Q}}}}_{{theo}}}{{\mbox{=}}}\frac{{{{{{\rm{N}}}}}}\,{{\times\, }}{{{{{\rm{n}}}}}}}{({{{{{\rm{J}}}}}}\times {{{{{\rm{t}}}}}})/({{{{{\rm{q}}}}}}\times {{{{{{\rm{N}}}}}}}_{{{{{{\rm{A}}}}}}})}\times 100\%$$

Where φ_*F*_ is the Faradaic efficiency of H_2_, Q_*exp*_ is the number of products measured from gas chromatography (GC, Agilent 7890B), Q_*theo*_ is the maximum amount of products that can be expected from the passed current, N (mol) is the number of products recorded in GC during a period of reaction time, n is the number of electrons transferred in the electrochemical reaction, J (mA) is the passed photocurrent, q (1.6 × 10^−19^ C) is the elementary charge, and N_*A*_ (6.02 × 10^23^) is the Avogadro constant.

The IPCE was measured under monochromatic illumination from a 150 W Xe lamp (Zolix LSH-X150) equipped with a monochromator (Omni-300) at 0 V vs. RHE according to Eq. (4):

4 $${{\mbox{IPCE (}}}\%{{\mbox{)=}}}\frac{{{{{{\rm{J}}}}}}\times 1240}{{{{{{\rm{P}}}}}}\times \lambda }\times 100\%$$

Where J (mA/cm^2^) is the photocurrent density, P is the incident illumination intensity (100 mW/cm^2^ in this work), and λ (nm) is the incident light wavelength.

The minority carrier lifetime was measured by the photoconductance lifetime tester (Sinton Instruments, WCT-120) at room temperature (25–30 °C). Before testing, the instrument WCT-120 has been turned on for at least 30 min to reach a stable internal temperature. The pyramid pn^+^-Si is centered on the instrument with the n^+^ type Si facing up. The height of the flash is about 45 cm above the sample. The analysis was conducted in quasi-steady-state mode. The carrier diffusion length was calculated according to Eq. (5):

5 $${{\mbox{L}}}p\,{{\mbox{(m)=}}}\sqrt{{\tau }_{p}\times {{{{{{\rm{D}}}}}}}_{p}}$$

Where τ_*p*_ is the minority carrier lifetime of n-type Si, which is above 11 µs in our study, and D*p* (12.4 cm^2^/s at 298 K) is the diffusion coefficient of the hole.

### CFD method

Comsol Multiphysics 5.5 was used to carry out calculations with surface tension dominant two-phase flows^58^. The wettability of the electrode was varied in the simulations by varying the contact angle of the material for a hydrogen-water system. The momentum equations for the entire flow field are given as Eqs. (6–9):

6 $$\rho \times \frac{\partial u}{\partial t}+\rho \times (u\times \nabla )\times u=\nabla \times [-P+\tau ]+F+\rho \times g$$

7 $$\rho \times \nabla \times {{{{{\rm{u}}}}}}{{\mbox{=}}}0$$

8 $$\tau={\mu }\times (\nabla \times {{{{{\rm{u}}}}}}+{(\nabla \times {{{{{\rm{u}}}}}})}^{{{{{{\rm{T}}}}}}})$$

9 $${{{{{\rm{F}}}}}}=\sigma \times k\times n\times \delta$$

Where *ρ* (kg/m^3^) is the density, *u* (m/s) is the velocity components in *y* direction, *t* (s) is the time, *P* (Pa) is the pressure, *τ* is the deviatoric stress tensor for a Newtonian fluid, *F* (N/m^3^) is the surface tension force per unit volume, *g* (m/s^2^) is the acceleration due to gravity, *μ* (N ∙ s/m^2^) is the dynamic viscosity, *T* (K) is the temperature (293.1 K in this work), *σ* (N/m) is the surface tension coefficient (0.072 N/m in this work), *k* (m^−1^) is the interfacial curvature, n is the interfacial unit normal vector, and *δ* is the delta function centered at the interface.

## Supplementary information

Supplementary Information

Description of Additional Supplementary Files

Supplementary Movie 1

Supplementary Movie 2

## Data Availability

The authors declare that all data supporting the results of this study are available within the paper and its supplementary information files or from the corresponding author upon reasonable request. Source data are provided with this paper.

## Source data

Source Data
