# Supplementary material for: Stable solar water splitting with wettable organic-layer-protected silicon photocathodes
Source: Nat Commun. 2022 Aug 1;13:4460. doi: 10.1038/s41467-022-32099-1 (PMC9343433; doi:10.1038/s41467-022-32099-1)
Supplement: Supplementary file 1 — Supplementary Information [file 41467_2022_32099_MOESM1_ESM.pdf]

Supplementary Information

Stable Solar Water Splitting with Wettable Organic-layer-protected Silicon Photocathodes

Bo Wu,[1,2§] Tuo Wang,[1,2§] Bin Liu,[1,2] Huimin Li,[1,2] Yunlong Wang,[1,2] Shujie Wang,[1,2] Lili Zhang,[1,2] Shaokun Jiang,[3] Chunlei Pei,[1,2] and Jinlong Gong[*1,2,4]

[1]*School of Chemical Engineering and Technology, Tianjin University, Tianjin 300072, China.*

[2]*Key Laboratory for Green Chemical Technology of Ministry of Education, Tianjin University, Tianjin 300072, China.*

[3]*Purification Equipment Research Institute of Handan, Handan, Hebei 056000, China.*

[4]*Joint School of National University of Singapore and Tianjin University, International Campus of Tianjin University, Binhai New City, Fuzhou 350207, China.*

[§] These authors contributed equally to this work.

*Corresponding Author: jlgong@tju.edu.cn.

Contents

1. Supplementary Figures and Tables

2. Supplementary References

Supplementary Figures and Tables

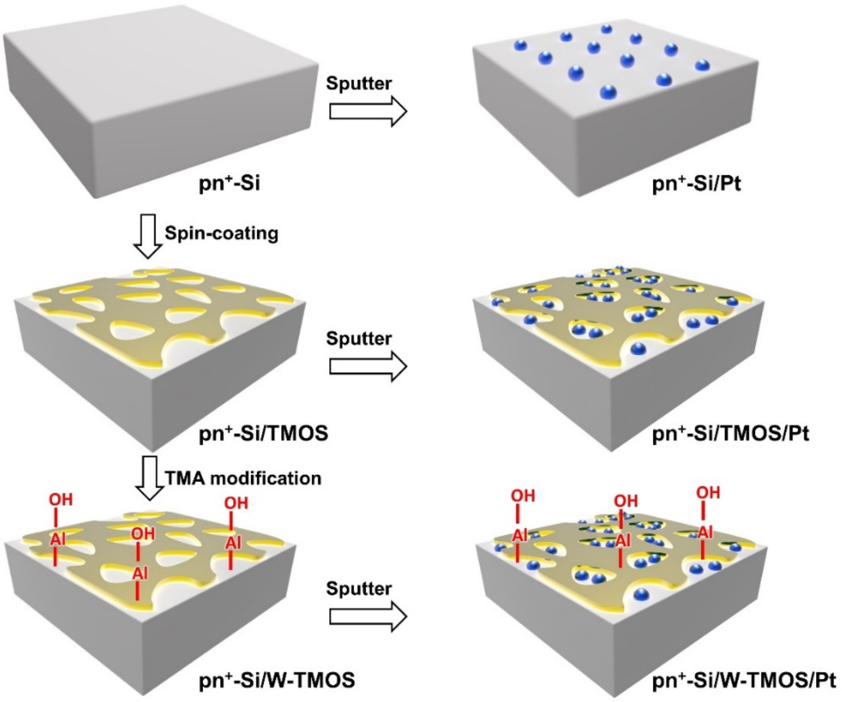

Supplementary Fig. 1 Schematic diagram of the preparation steps for different photocathodes with different protective layers.

We adopted an easy spin-coating method to deposit the hydrophobic TMOS layer above the pn^+-Si surface. To compare with the hydrophobic layer, hydrophobic layer was modified by TMA adsorption followed by ambient exposure, which could be converted into hydrophilic hydroxyl groups. After that, the Pt nanoparticles were sputtered above the electrode surface. Clusters of Pt were sputtered both on top of the TMOS layer as well as in the pores of the TMOS layer. In our illustration, we only show the Pt in the pores to focus on the point that the Si/Pt ohmic contact in the pores of the discontinuous organic layer facilitates improved electron transfer.

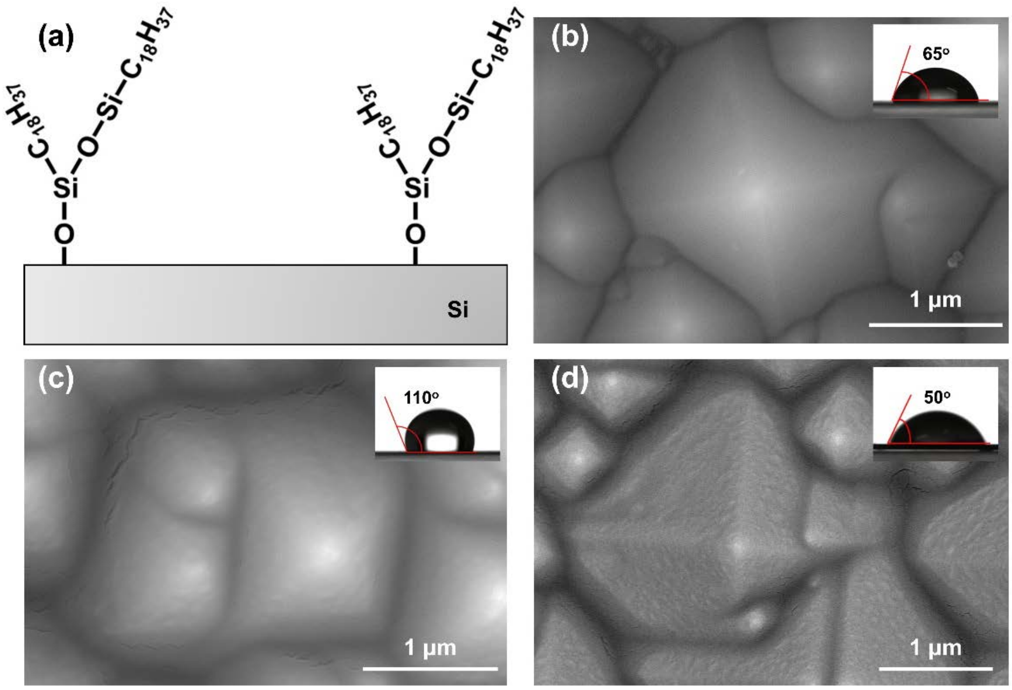

Supplementary Fig. 2 Surface morphology of pyramid pn$^+$-Si based photocathodes. (a) Schematic diagram of Si/TMOS. Top view SEM image of pyramid (b) pn$^+$-Si, (c) pn$^+$-Si/TMOS, and (d) pn$^+$-Si/W-TMOS. (Inset: contact angle.)

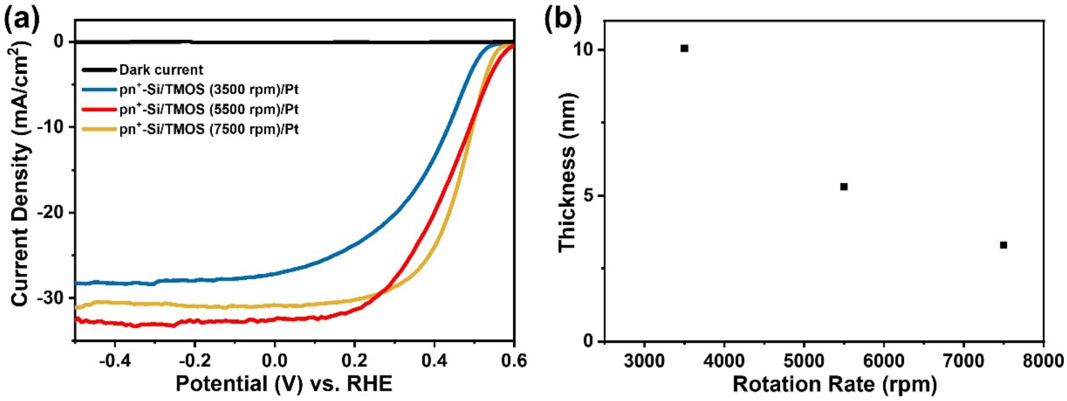

Supplementary Fig. 3 Impact of the rotation rate on the PEC performance. (a) J-V curves of photocathodes with different thicknesses of TMOS layer. (b)The thickness of the TMOS layer at different rotation rates.

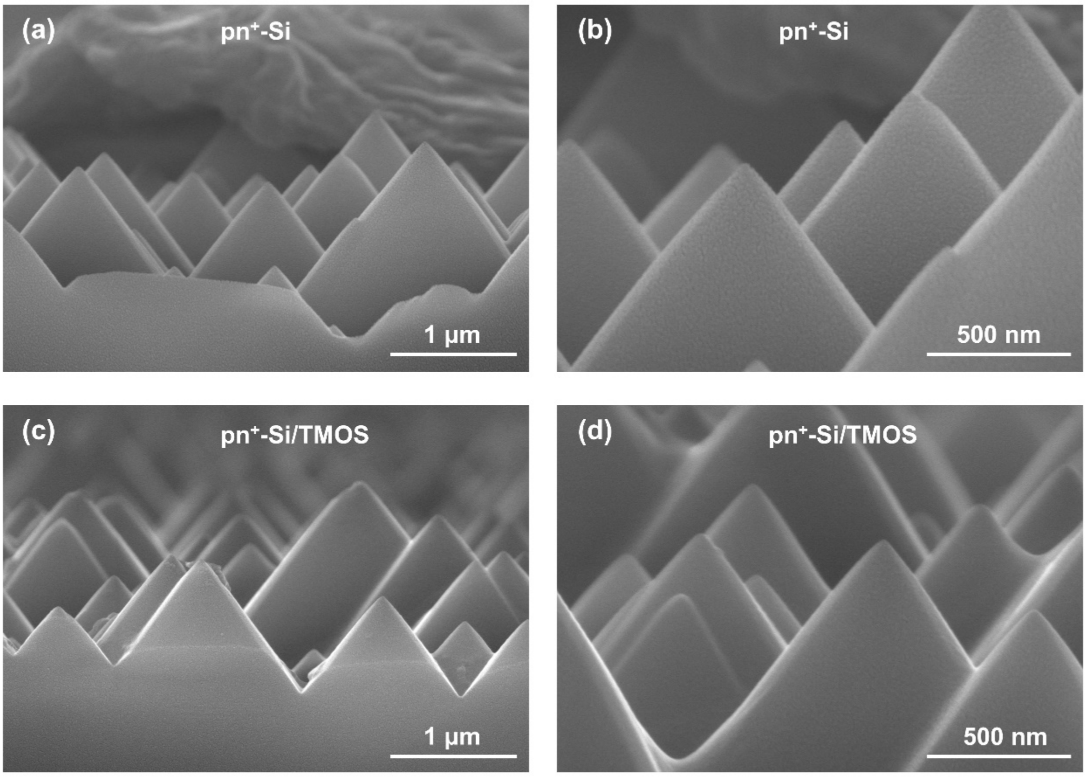

Supplementary Fig. 4 Side view SEM of pyramid electrodes. Side view SEM images of (a, b) pyramid pn^+-Si and (c, d) pyramid pn^+-Si/TMOS.

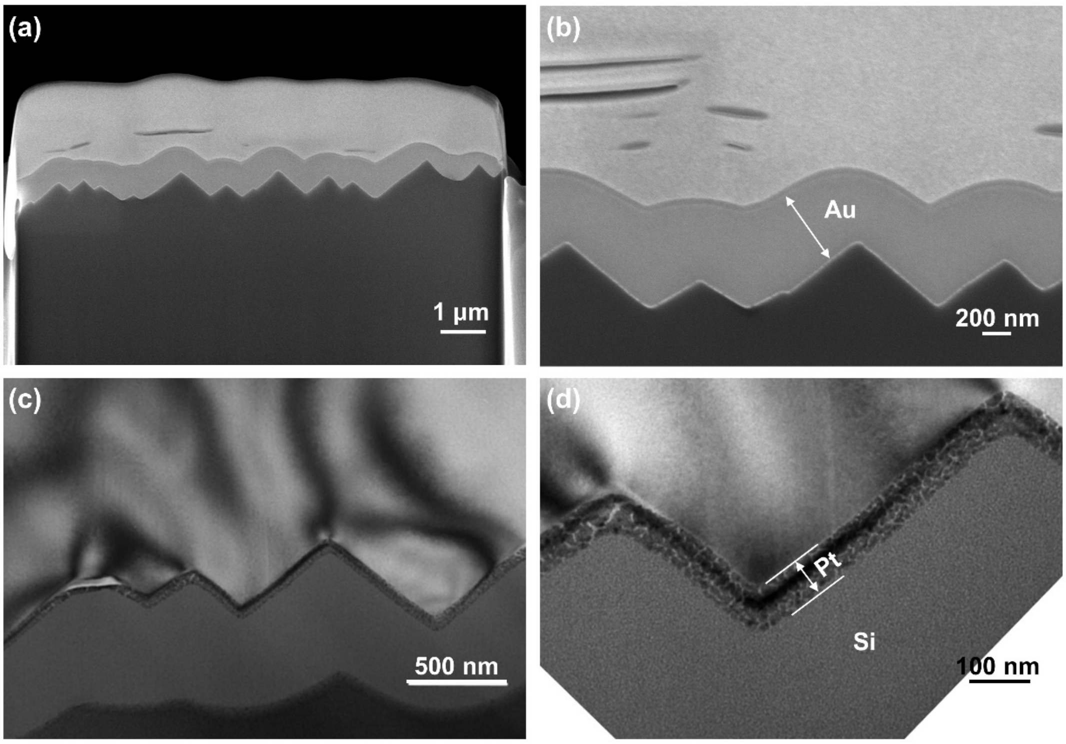

Supplementary Fig. 5 Si/TMOS interfacial structure. SEM images of (a, b) pyramid pn$^+$-Si/TMOS after the FIB and (c, d) Cross-section TEM images of pn$^+$-Si/TMOS.

The thin organic TMOS layer may be destroyed during the after the focus ion beam (FIB) for sample preparing. The images only show a thick Pt layer which was sputtered above the TMOS layer to increase the conductivity of the sample.

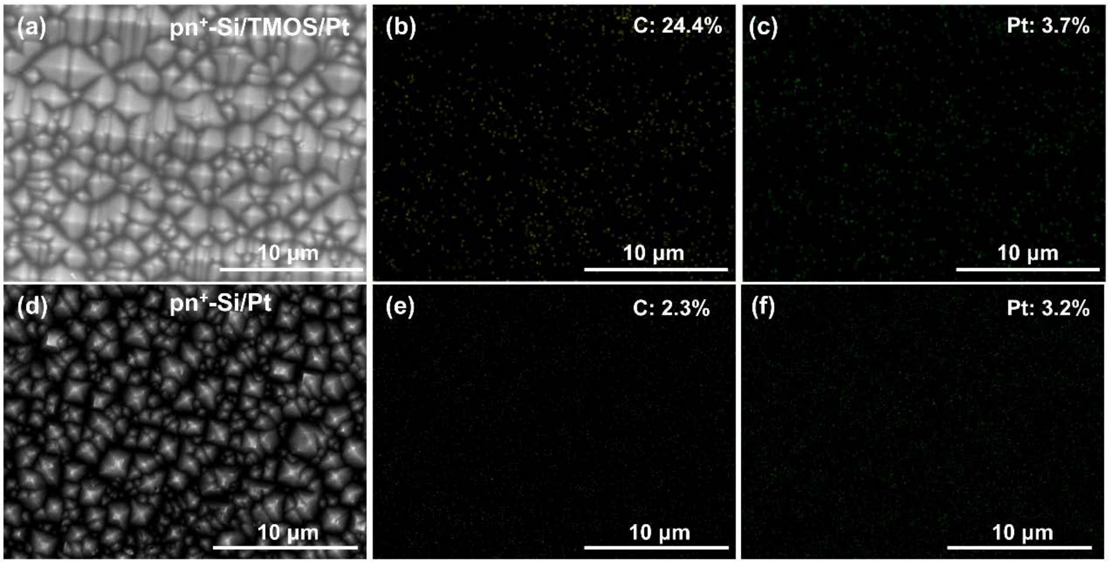

Supplementary Fig. 6 Elemental distribution of pyramid pn$^+$-Si based photocathodes. SEM images of (a, d) pyramid pn$^+$-Si/TMOS/Pt and pyramid pn$^+$-Si/Pt, and the corresponding EDS element mapping of C and Pt for (b, c) pyramid pn$^+$-Si/TMOS/Pt and (e, f) pyramid pn$^+$-Si/Pt

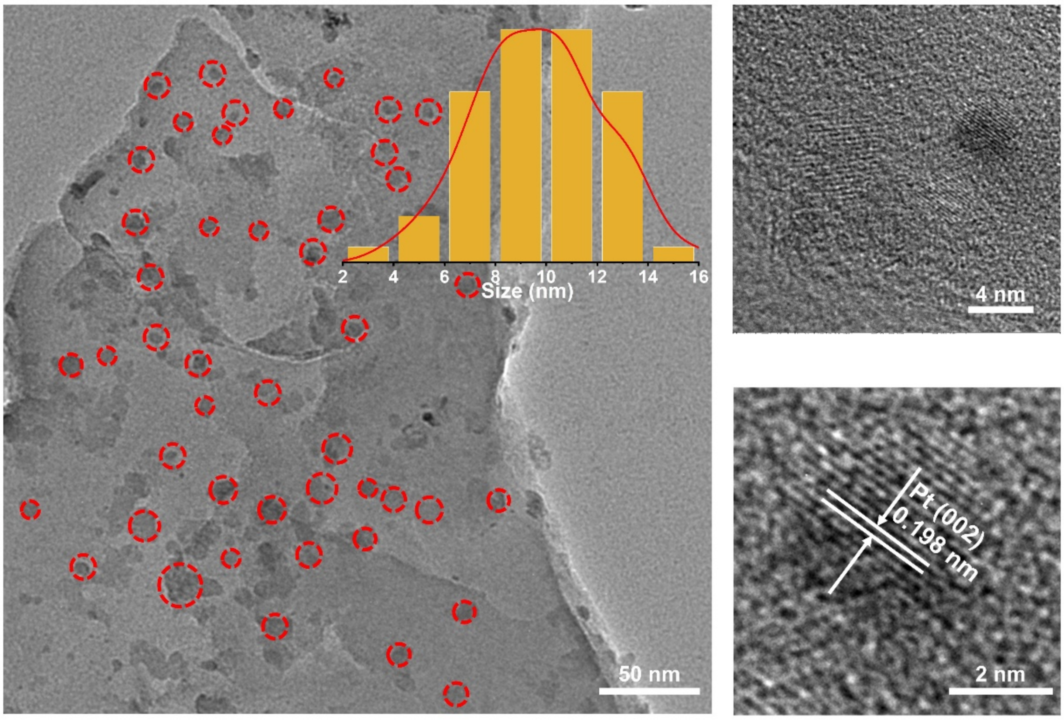

Supplementary Fig. 7 TEM results of pyramid pn$^+$-Si/TMOS/Pt (Inset: Pt particles size distribution).

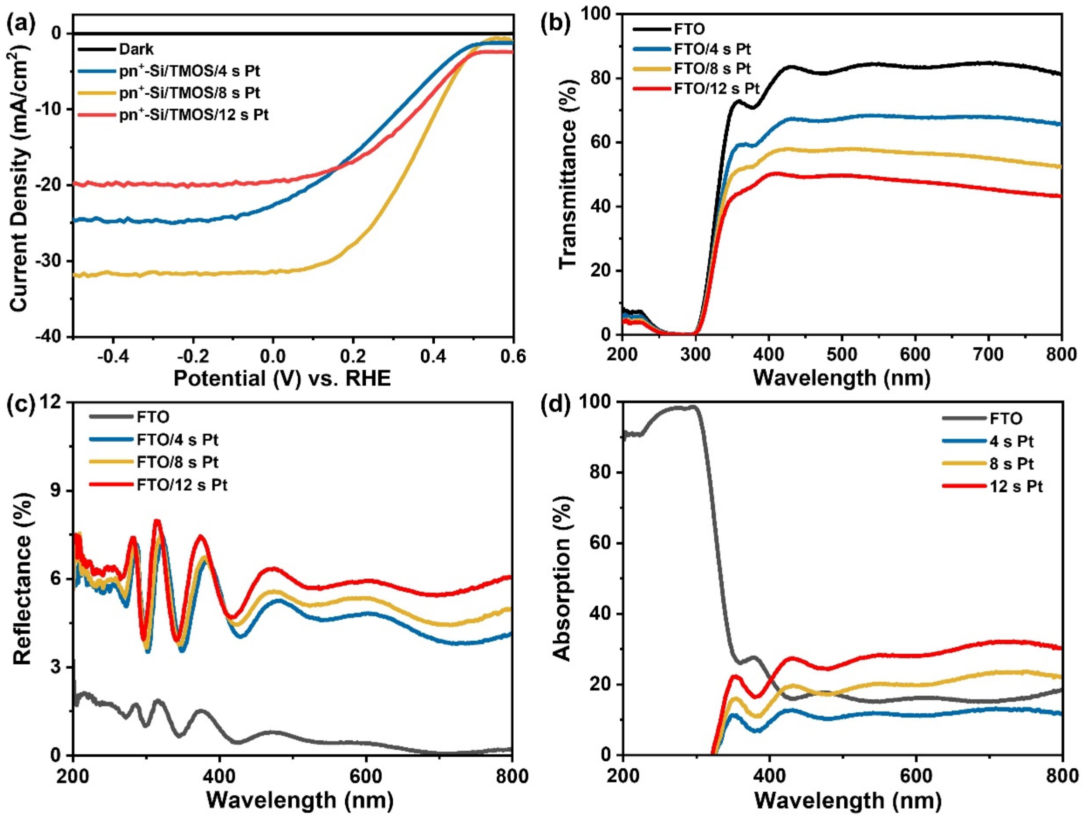

Supplementary Fig. 8 PEC performance of photocathodes with different loading amount of Pt. (a) J-V curves of photocathodes with different loading amounts of Pt. (b) Transmittance spectra, (c) Reflectance spectra, and (d) Absorption spectra of fluorine-doped tin dioxide (FTO) and FTO with different loading amounts of Pt.

The PEC performance of the photocathode is determined by both the light absorption efficiency of the semiconductor and the utilization of the charger carrier by the co-catalyst. When the sputtering time is 4 s, a less active site was obtained for the HER. Furthermore, Pt particles may be hard to contact the electrolyte, making it hard to transfer the electron. Thus, a lower saturation photocurrent was obtained. However, when the sputtering time was increased to 12 s, the parasitic optical loss caused by the Pt layer also leads to a bad PEC performance. We have added the related description in the revised SI (Fig. S8).

$$\text{Absorption}_{(FTO)} = 100 - \text{Transmittance}_{(FTO)} - \text{Reflectance}_{(FTO)}$$

$$\text{Absorption}_{(Pt)} = 100 - \text{Transmittance}_{(Pt)} - \text{Reflectance}_{(Pt)} - \text{Absorption}_{(FTO)}$$

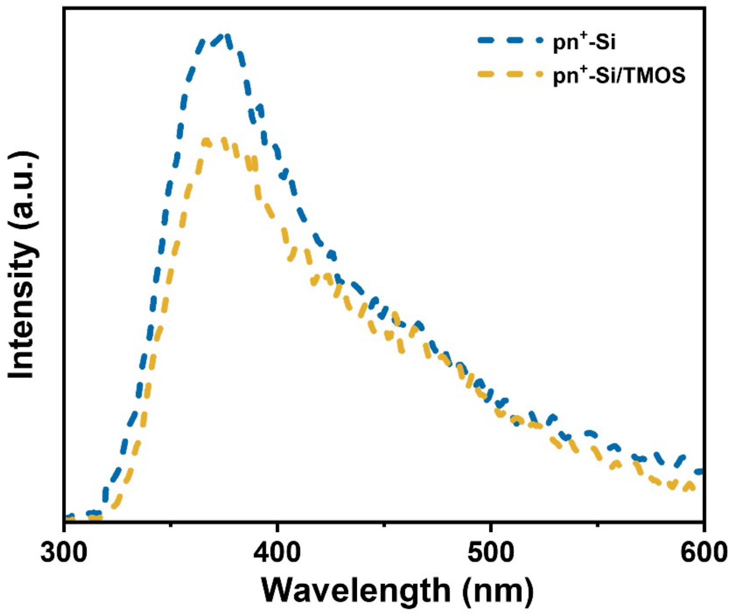

Supplementary Fig. 9 PL spectra of pyramid pn$^+$-Si and pn$^+$-Si/TMOS.

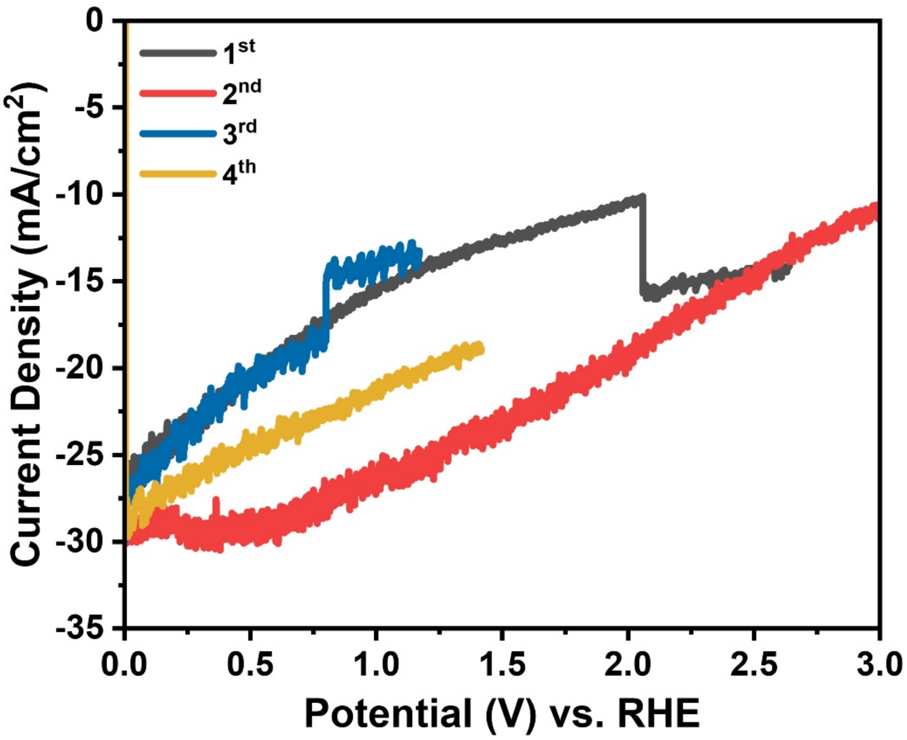

Supplementary Fig. 10 Stability of four pyramid pn$^+$-Si/Pt samples from different batches.

To thoroughly evaluate the stability of the unmodified electrodes to understand the natural variation in lifetime, four pyramid pn$^+$-Si/Pt samples from different batches were measured. All results reveal that the pyramid pn$^+$-Si/Pt sample decays quickly within 3 h.

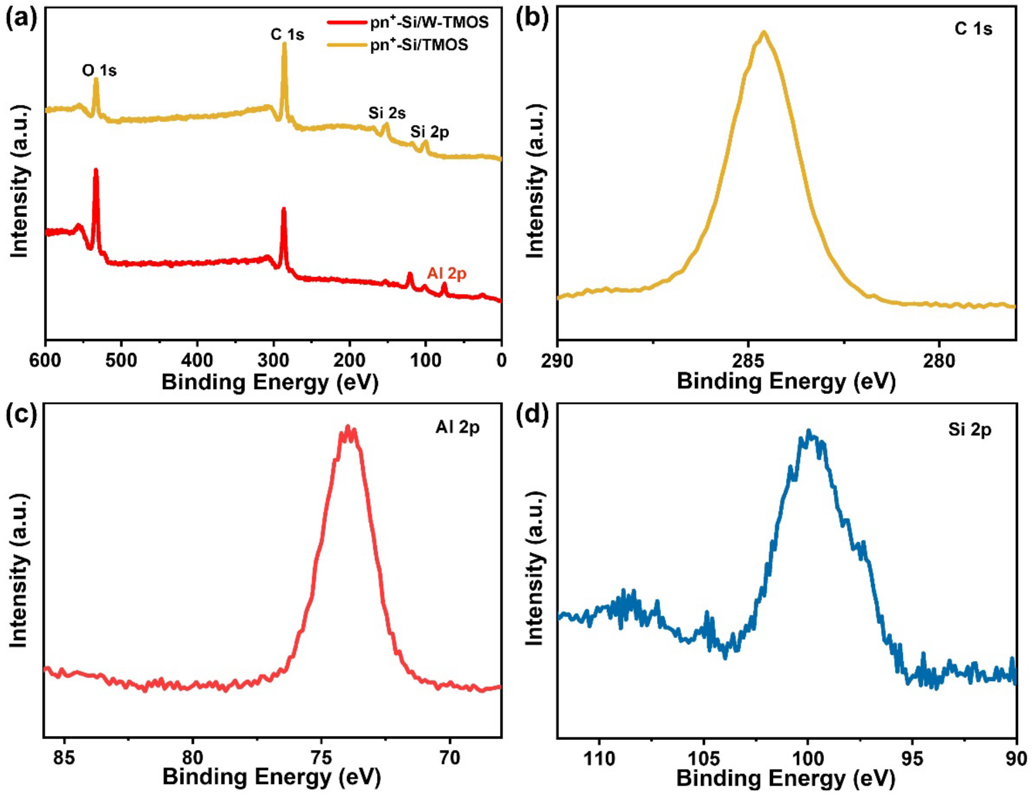

Supplementary Fig. 11 XPS of pn$^+$-Si/TMOS and pn$^+$-Si/W-TMOS. (a) XPS spectra of pyramid pn$^+$-Si/W-TMOS and pn$^+$-Si/W-TMOS. (b) C 1s, (c) Al 2p, and (d) Si 2p of pn$^+$-Si/W-TMOS.

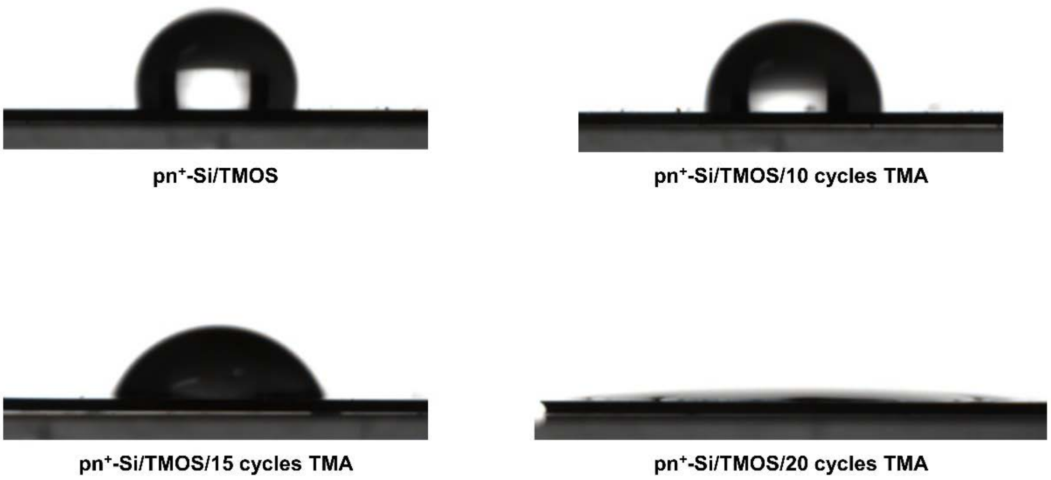

Supplementary Fig. 12 Contact angles of electrodes with different dosing cycles of TMA.

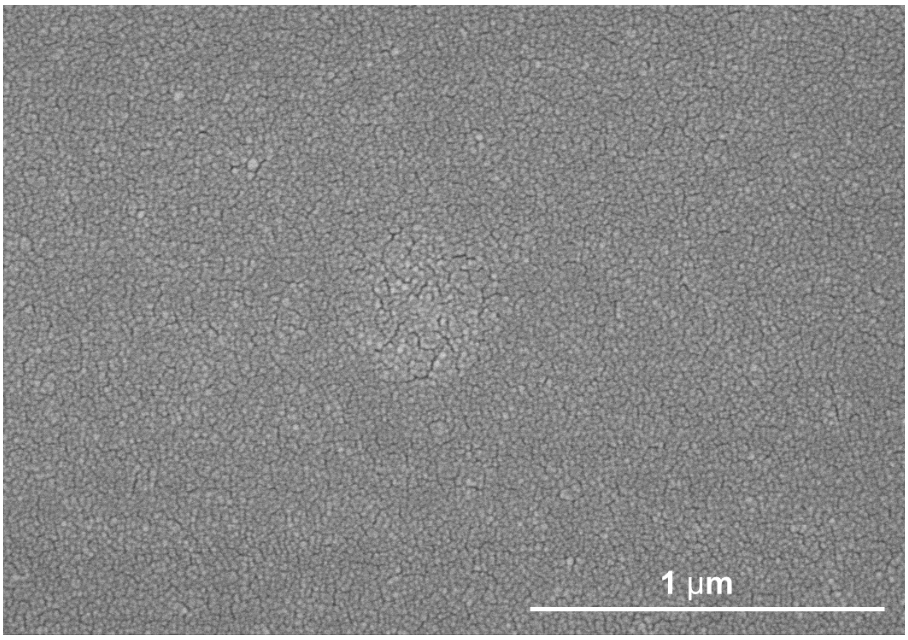

Supplementary Fig. 13 SEM image of the planar p^{++}-Si/W-TMOS sample.

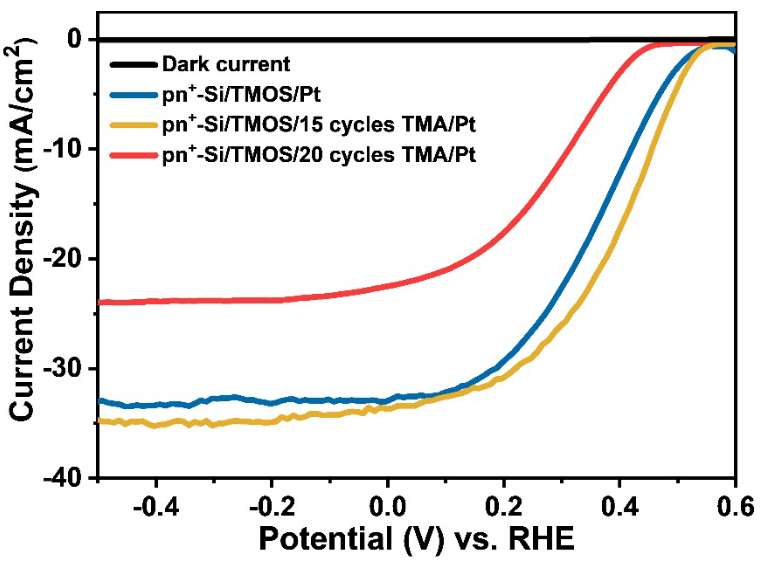

Supplementary Fig. 14 J-V curves of photocathodes with different dosing cycles of TMA.

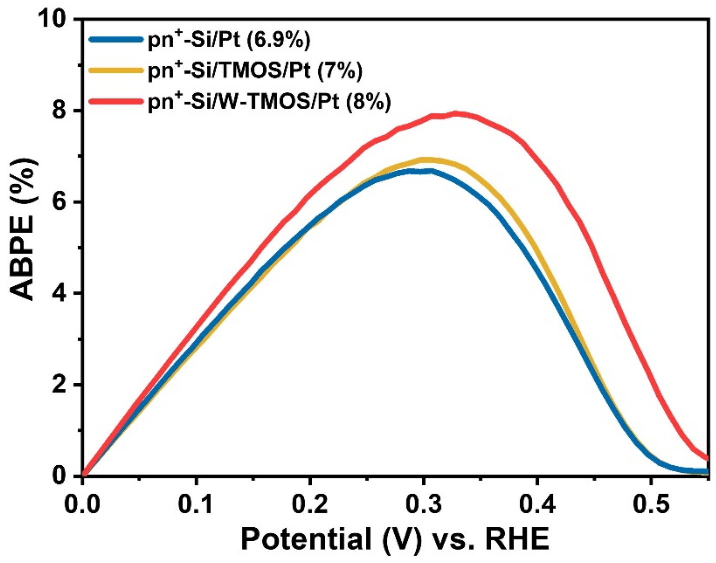

Supplementary Fig. 15 ABPE curves of pyramid pn$^+$-Si/W-TMOS/Pt, pn$^+$-Si/TMOS/Pt, and pn$^+$-Si/Pt photocathodes.

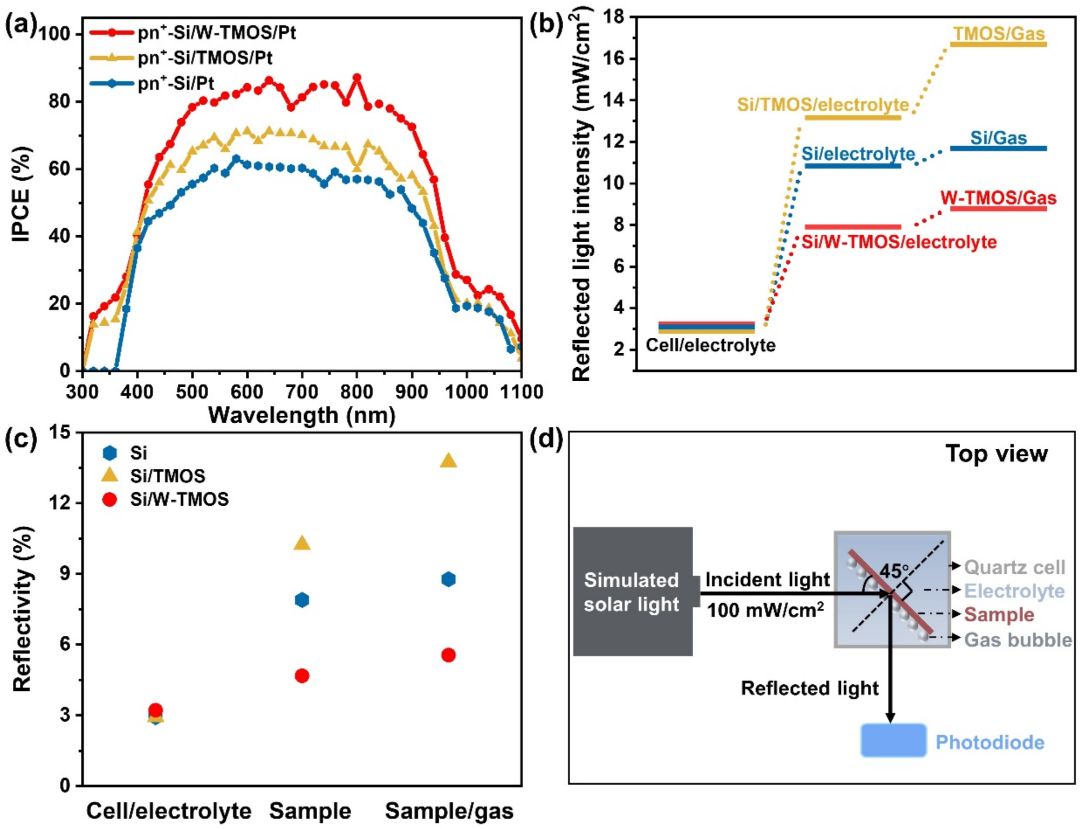

Supplementary Fig. 16 Reflection of incident light associated with bubble evolution on different photocathode surfaces. (a) IPCE curves, (b) Reflected light intensity, (c) Reflectivity of photocathodes with different protective layer and gas evolving conditions (Cell/electrolyte: baseline reflectivity without sample; Sample: sample only without gas evolving; Sample/gas: samples with gas evolving at the same current density of 30 mA/cm^2). (d) Schematic illustrations of the set up for the testing the reflected light intensity.

The reflectivity of different interface was obtained by the equation:

$$\text{Reflectivity} = \frac{\text{Reflected light intensity}_{(cell\ \&\ sample)} - \text{Reflected light intensity}_{(cell)}}{100} \times 100\%$$

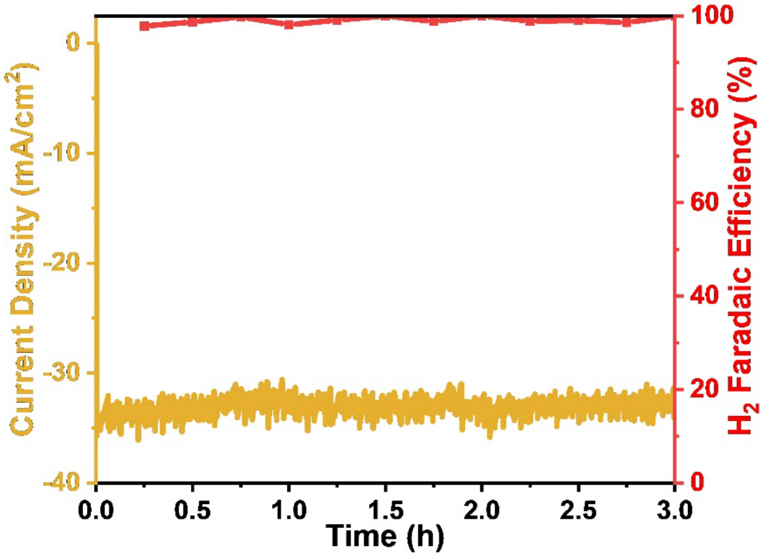

Supplementary Fig. 17 Time dependent Faradaic efficiency for H_2 of pyramid pn^+-Si/W-TMOS/Pt sample at 0 V vs. RHE.

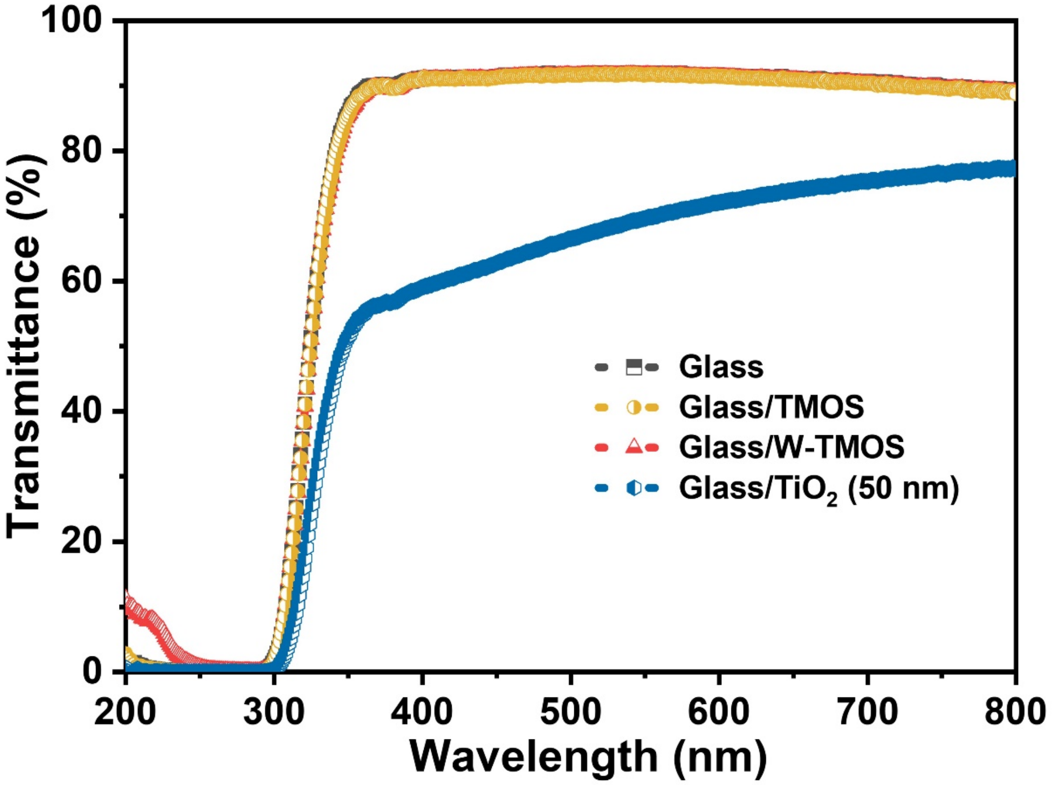

Supplementary Fig. 18 Transmittance of W-TMOS layer, TMOS layer, and TiO_2 (50 nm).

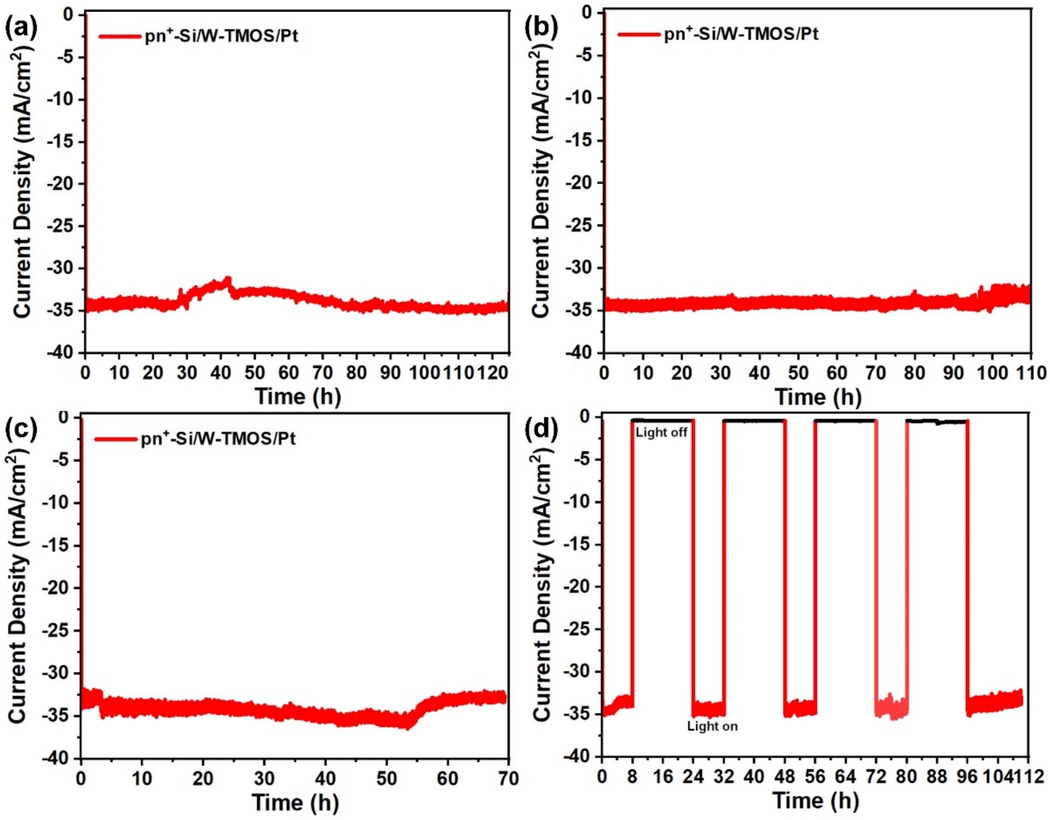

Supplementary Fig. 19 Stability of pyramid pn$^+$-Si/W-TMOS/Pt. (a), (b), (c) Stability of three pyramid pn$^+$-Si/W-TMOS/Pt samples from different batches under 0 V vs. RHE. (d) Stability test in simulated realistic system (8 h simulation and 16 h dark) under −0.4 V vs. RHE.

To thoroughly evaluate the stability of the photocathodes protected by W-TMOS, three pyramid pn$^+$-Si/W-TMOS/Pt samples from different batches were measured. All results reveal that the pyramid pn$^+$-Si/W-TMOS/Pt samples could achieve a long-term stability above 110 h.

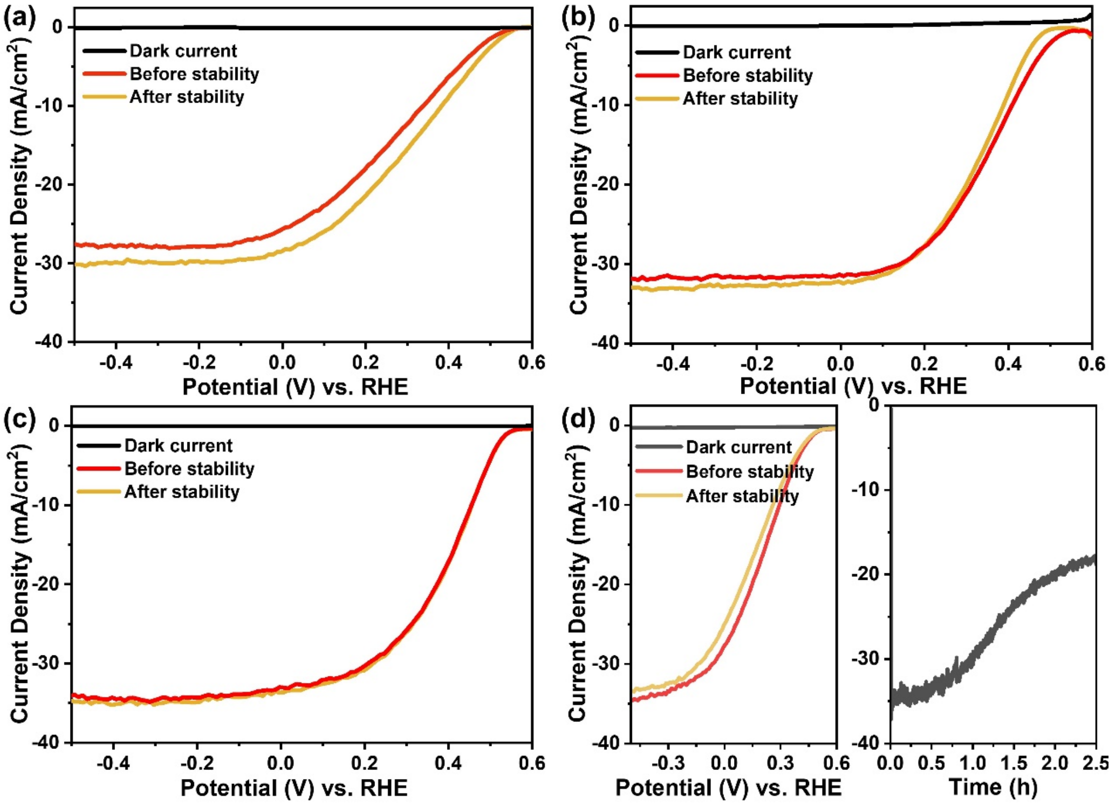

Supplementary Fig. 20 J-V curves of photocathodes with different protective layer before/after stability tests. J-V curves before/after stability test of pyramid (a) pn$^+$-Si/Pt, (b) pn$^+$-Si/TMOS/Pt, and (c) pn$^+$-Si/W-TMOS/Pt. (d) J-V curves before/after stability test, and stability test of pn$^+$-Si/TMOS (HT)/Pt at 0 V vs. RHE.

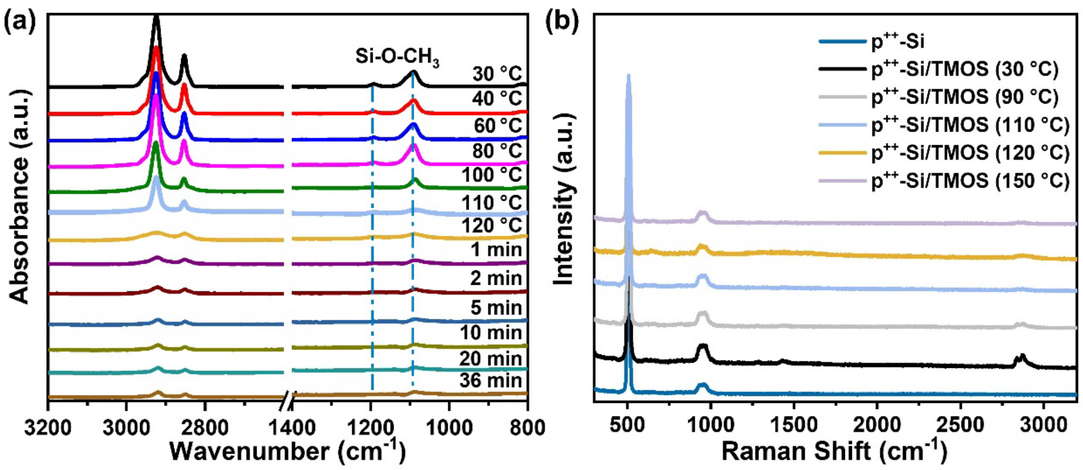

Supplementary Fig. 21 Chemical structure changes of TMOS layer during the baking process. (a) Quantitative *in situ* DRIFTS spectroscopy of Si/TMOS of different temperatures. (b) Raman spectroscopy recorded from Si/TMOS (different temperatures).

Supplementary Fig. 12a shows the reduced peak at 1088 and 1194 cm^{-1}, which indicates the hydrolysis and condensations of TMOS with the increased temperature. At the same time, the reduced peak at 2851 and 2924 cm^{-1} is due to the evaporation of ethanol in the precursor.

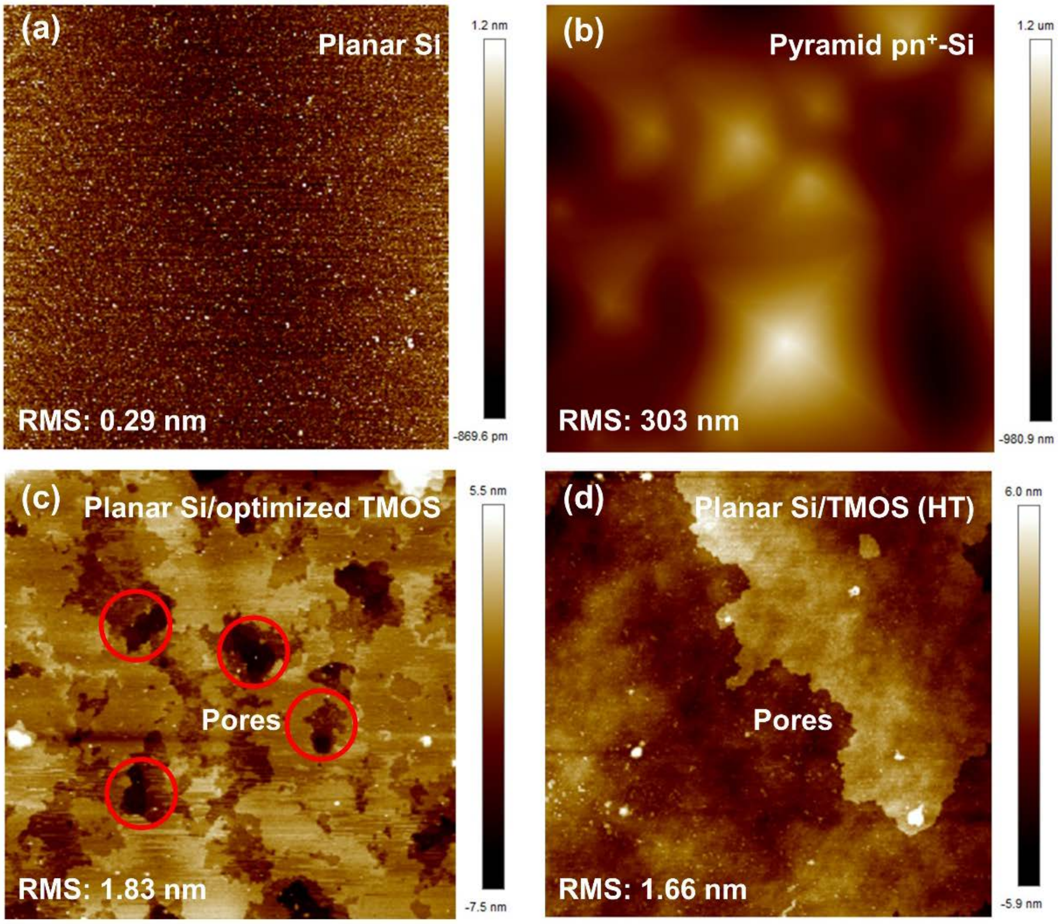

Supplementary Fig. 22 AFM of TMOS layer under different baking temperature. AFM images of (a) planar p^{++}-Si, (b) pyramid pn^{+}-Si, (c) p^{++}-Si/TMOS, and (d) p^{++}-Si/TMOS (HT). The scan size is 5 µm × 5 µm.

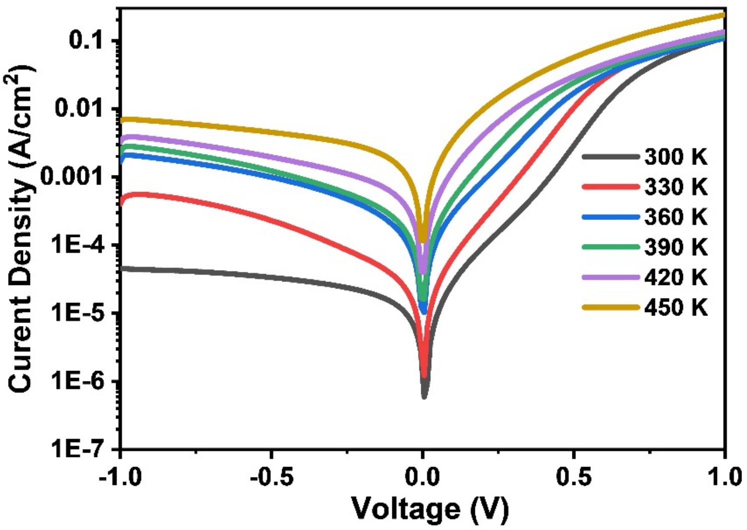

Supplementary Fig. 23 Dark solid J-V curves of pyramid pn$^+$-Si/TMOS/Pt at different temperatures.

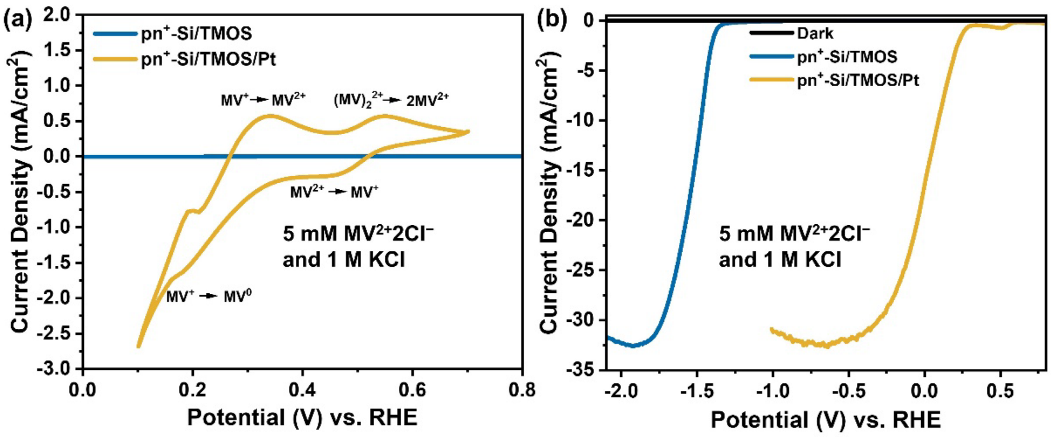

Supplementary Fig. 24 Pt particles effects towards the electron transfer efficiency. (a) Cyclic voltammogram, (b) J-V curves of pyramid pn^+-Si/TMOS and pn^+-Si/TMOS/Pt using aqueous methyl-viologen as the redox couple (5 mM $MV^{2+}2Cl^-$ and 1 M KCl). The scan rate is 50 mV/s.

The methyl violet used in the test is brought from Sigma-Aldrich.

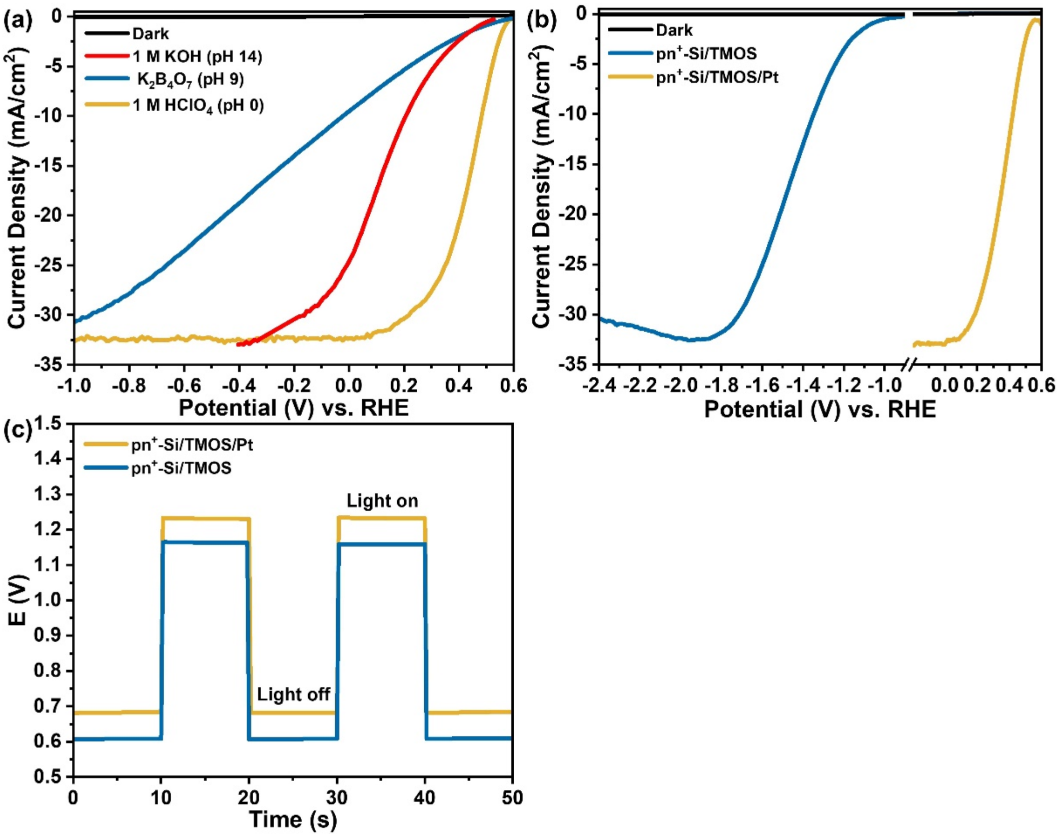

Supplementary Fig. 25 The effects of Pt particles on the photovoltage of photocathodes. (a) The J-V curves of pyramid pn$^+$-Si/TMOS/Pt under different solutions with different pH. (b) The J-V curves of pyramid pn$^+$-Si/TMOS/Pt and pn$^+$-Si/TMOS in $HClO_4$. (c) The open circuit potential versus time of pyramid pn$^+$-Si/TMOS/Pt and pn$^+$-Si/TMOS samples in the dark and under AM 1.5G illumination, in 1 M $HClO_4$ (pH 0) electrolyte.

The photovoltage was determined from the difference of open circuit potentials of the sample in the dark and under illumination. The open circuit potential was measured by monitoring the resting potential of samples (vs. the reference electrode) in the open circuit condition, with the illumination turned on and off manually. The duration of illumination on and off were both 10 s, respectively.

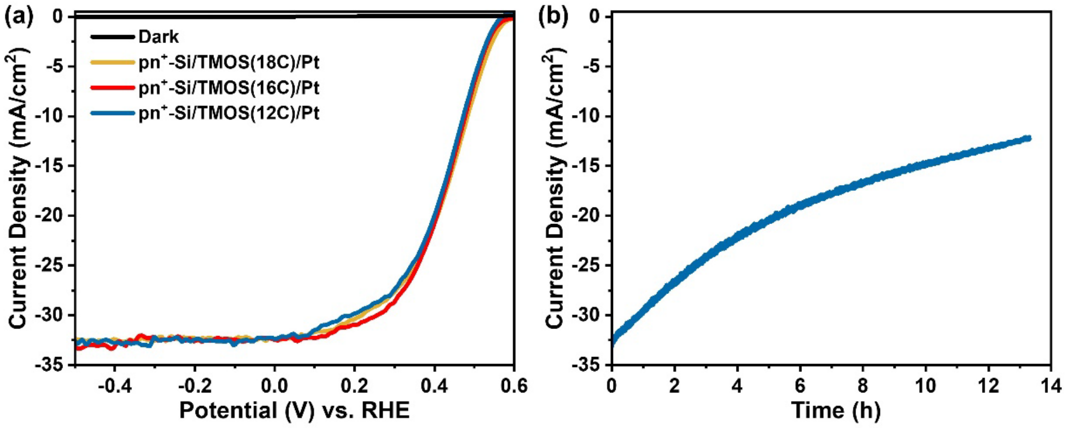

Supplementary Fig. 26 PEC performance of photocathode protected by organic protective layers with different chain length. (a) J-V curves of pyramid pn^+-Si based photocathodes protected by different organic protective layer with different chain length. (b) Stability test of pn^+-Si/TMOS(12C)/Pt at 0 V vs. RHE.

The method used to modify hexadecyltrimethoxysilane ($CH_3(CH_2)_{15}Si(OCH_3)_3$, Shanghai Meryer Chemical Technology Co., Ltd.), which is defined as TMOS(16C) and dodecyltrimethoxysilane ($CH_3(CH_2)_{11}Si(OCH_3)_3$, Shanghai Meryer Chemical Technology Co., Ltd.), which is defined as TMOS(12C) is same with the method used for the TMOS layer described in the manuscript.

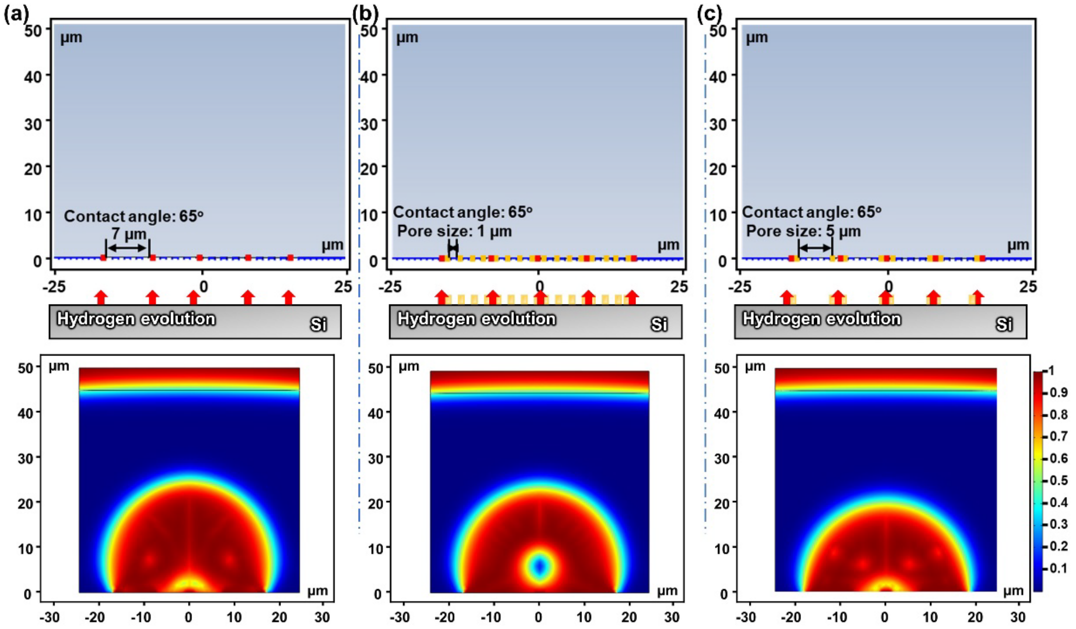

Supplementary Fig. 27 CFD simulations for electrode-electrolyte interface. Simulation models, parameter settings, schematic illustrations, and simulation result of Si-liquid contact for planar (a) Si, (b) Si/TMOS, and (c) Si/TMOS (HT).

We adopted constant H_2 flow streams in the model to represent the hydrogen evolution reaction. In these models, the blue line represents the electrode/electrolyte interface. The hydrogen gas evolving site, which was defined as a H_2 gas inlet in the CFD model, was set to 1 μm, and the flow rate of H_2 is determined according to the photocurrent density. Furthermore, the length of a single hydrophobic segment was also set to 1 μm. The distance between the adjacent yellow bars is the pore size of the TMOS layer. As a result, the blue bars in between yellow bars represent the exposed Si at pores. The grey-blue background represents the electrolyte, which is blue in the simulation result.

Different contact angles were used for different segments when simulating the Si/liquid contact among the different electrodes with different structures (Fig. 3, S27, S28). The hydrophobic segment representing the TMOS region was set as 110°, while the hydrophilic segment was set as 65°.

Since the electrode is symmetrical, only half of the electrode was simulated in the CFD software, while the other half was represented using the symmetric boundary condition. We presented the simulation results of the entire electrode for clarity purposes.

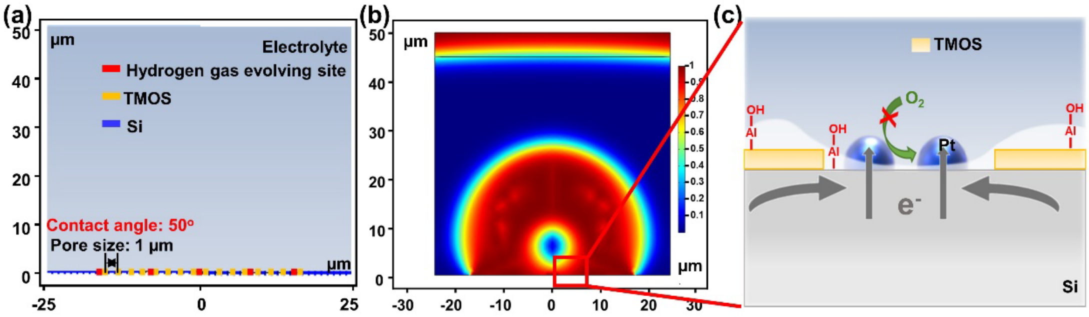

Supplementary Fig. 28 CFD simulations for electrode-electrolyte interface of Si/W-TMOS. (a) Simulation model and parameter setting, (b) Simulation result, and (c) Corresponding schematic illustration (not to scale) of the electron conduction path for planar Si/W-TMOS. The color bar shows the volume fraction of H_2 gas in the liquid/gas mixture.

Constant H_2 flow streams were used for representing the hydrogen evolution reaction in the model. The red bar is defined as the H_2 gas inlet in the CFD model, which represents the H_2 gas evolving sites of the electrode. In addition, the flow rate of H_2 is set according to the photocurrent density. In the model, the blue line represents the electrode/electrolyte interface. The yellow bar is defined as the TMOS layer, whose contact angle is 110°, while the blue bar is defined as the exposed Si, whose contact angle is 50°. The grey-blue background is defined as the electrolyte, which is blue in the simulation result.

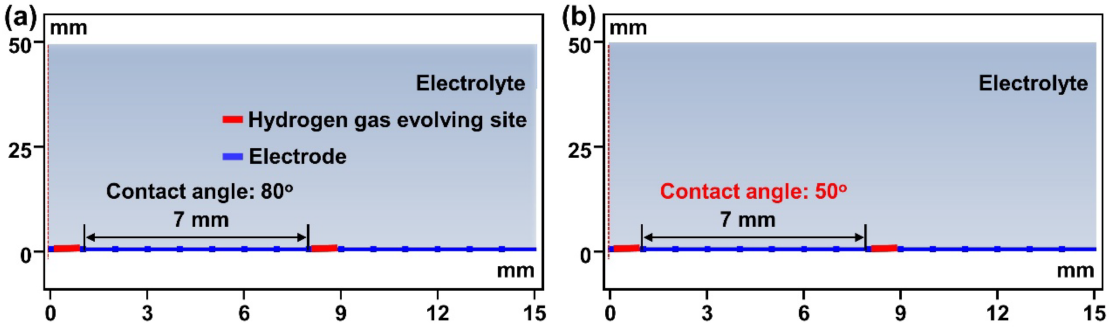

Supplementary Fig. 29 Simulation models of different electrodes for bubble growth. Simulation models and parameter settings for planar (a) Si/TMOS and (b) Si/W-TMOS.

The surface wettability of the Si/TMOS or Si/W-TMOS electrode was obtained from the contact angle test of the pn$^+$-Si/TMOS/Pt or pn$^+$-Si/W-TMOS/Pt photocathode. The contact angle of the entire system was used when simulating the bubble growth among the different electrodes with different wettability. The model with a contact angle of 80° was used to represent the Si/TMOS/Pt electrode, while the model with a contact angle of 50° was used to represent the electrode protected by the W-TMOS layer. In these models, H_2 flow streams were added to represent the hydrogen evolution reaction, and the flow rate was determined according to the photocurrent density. The blue line in the model represents the electrode/electrolyte interface, where the red bar is defined as the H_2 gas inlet, representing the H_2 gas evolving sites. Furthermore, the grey-blue background was used to represent the electrolyte, which is blue in the simulation results in Fig. 4 of the manuscript.

Supplementary Table 1

Comparison of the PEC performance for pn$^+$-Si-based photocathodes protected by different protective layers

Photocathode	Deposition method	Stability/ Current density	References
pn$^+$-Si/Ti/TiO_2/Pt	Sputtering	72 h (22 mA/cm^2) (λ>635 nm)	J. Am. Chem. Soc. 2013[1]
p-Si/$SrTiO_3$/Ti/Pt	Molecular beam epitaxy	35 h (35 mA/cm^2)	Nature. Nanotech. 2014[2]
pn$^+$-Si/Mo/MoS_2	/	100 h (20 mA/cm^2)	Adv. Energy. Mater. 2014[3]
pn$^+$-Si/Al_2O_3/Pt	ALD	42 h (30 mA/cm^2)	Appl. Phys. Lett. 2016[4]
p$^+$nn$^+$-Si/GaN/Pt	E-beam	110 h (38 mA/cm^2)	Nano. Lett. 2018[5]
pn$^+$-Si/Ta_2O_5/Pt	ALD	200 h (34.7 mA/cm^2)	Ind. Eng. Chem. Res. 2019[6]
**pn$^+$-Si/Pt**<br>**pn$^+$-Si/TMOS/Pt**<br>**pn$^+$-Si/W-TMOS/Pt**	**Spin-coating**	**3 h (30 mA/cm^2)**<br>**110 h (32 mA/cm^2)**<br>**110 h (35 mA/cm^2)**	**This work**

Supplementary Table 2

Table of electron transport mechanisms

**Transport Mechanisms**	**Current Density**
Tunneling through	$J \, \alpha \, V^2 \exp\left(\frac{-a}{V}\right)$
Ohmic contact	$J \, \alpha \, V \exp\left(\frac{-b}{T}\right)$
Ionic conduction	$J \, \alpha \, \frac{V}{T} \exp\left(\frac{-c}{T}\right)$

Supplementary References

1. Seger, B. et al. Using TiO_2 as a conductive protective layer for photocathodic H_2 evolution. *J. Am. Chem. Soc.* **135**, 1057-1064, (2013).
2. Ji, L. et al. A silicon-based photocathode for water reduction with an epitaxial $SrTiO_3$ protection layer and a nanostructured catalyst. *Nat. Nanotechnol.* **10**, 84-90, (2015).
3. Bae, D. et al. Protection of Si photocathode using TiO_2 deposited by high power impulse magnetron sputtering for H_2 evolution in alkaline media. *Sol. Energy Mater Sol. Cells* **144**, 758-765, (2016).
4. Fan, R. et al. Stable and efficient multi-crystalline n^+p silicon photocathode for H_2 production with pyramid-like surface nanostructure and thin Al_2O_3 protective layer. *Appl. Phys. Lett.* **106**, 013902, (2015).
5. Vanka, S. *et al.* High efficiency Si photocathode protected by multifunctional GaN nanostructures. *Nano Lett.* **18**, 6530-6537, (2018).
6. Wang, T. et al. Transparent Ta_2O_5 protective layer for stable silicon photocathode under full solar spectrum. *Ind. Eng. Chem. Res.* **58**, 5510-5515, (2019).
