# Supplementary material for: Stable solar water splitting with wettable organic-layer-protected silicon photocathodes
Source: Nat Commun. 2022 Aug 1;13:4460. doi: 10.1038/s41467-022-32099-1 (PMC9343433; doi:10.1038/s41467-022-32099-1)
Supplement: Supplementary file 2 — Description of Additional Supplementary Files [file 41467_2022_32099_MOESM2_ESM.pdf]

Description of Additional Supplementary Files

File Name: Supplementary Movie 1
Description: Video of the bubble growth during the stability test of the pn^+-Si/TMOS/Pt photocathode.

File Name: Supplementary Movie 2
Description: Video of the bubble growth during the stability test of the pn^+-Si/WTMOS/Pt photocathode.
